# Grit subcomponents are differentially associated with practice trajectories underlying expertise development

**DOI:** 10.1038/s41598-025-22533-x

**Published:** 2025-10-29

**Authors:** Dijana Cocić, Brady S. DeCouto, Bradley Fawver, Rhiannon L. Cowan, David T. Hendry, A. Mark Williams, Merim Bilalić

**Affiliations:** 1 School of Sport, Exercise and Rehabilitation, Northumbria University, Ellison Square, Newcastle upon Tyne, NE1 8ST UK; 2 Florida State University, Tallahassee, FL USA; 3 Walter Reed Army Institute of Research – West, Joint Base Lewis-McChord, Washington, USA; 4 Department of Neurosurgery, University of Utah, Salt Lake City, UT 84132 USA; 5 School of Sport, Health and Exercise, Loughborough University, Loughborough, UK; 6 School of Psychology, Northumbria University, Ellison Square, Newcastle upon Tyne, NE1 8ST UK

**Keywords:** Practice, Personality traits, Generalized additive models (GAM), Longitudinal study, talent identification, Training, Human behaviour, Ageing

## Abstract

Practice is essential for skill acquisition, yet the psychological traits driving changes that occur with practice remain unclear. We extend previous work that identified grit as a key psychological trait underpinning practice accumulation in football. More specifically, we examined whether similar patterns exist in alpine ski racing, a sport characterized by seasonal constraints and limited practice opportunities. We collected retrospective estimates of practice hours from *N* = 231 elite youth skiers in Austria and the USA aged 6 to 19 years, alongside current measures of grit’s subcomponents, Consistency of Interests (CI) and Perseverance of Effort (PE). We used Generalized Additive Models (GAM) to analyze nonlinear practice trajectories. CI was positively associated with practice accumulation during early developmental stages up to age 12 years. From age 12 onward, PE became the dominant factor associated with practice. Notably, grit had the most pronounced association on engagement in unstructured practice activities, such as self-training and free play. These findings suggest that grit might impact expertise indirectly by affecting how athletes engage in practice over time. Understanding the differentiated roles of CI and PE in practice accumulation offers valuable insights for talent development programs; we emphasize the importance of fostering psychological traits that support sustained commitment and resilience in athletes.

**Supplementary Information:**

The online version contains supplementary material available at 10.1038/s41598-025-22533-x.

## Introduction

Practice is widely recognized as a crucial component in the acquisition of expertise. However, the specific mechanisms underlying how practice is accumulated remain less understood. In our previous study examining elite youth football players^1^, we observed that the personality trait of ‘grit’ is associated with the acquisition of practice. Results indicated that the grit subscale Consistency of Interests (CI) drove practice accumulation during the early developmental stages, up to the age of 12, while the other subscale, Perseverance of Effort (PE), became the dominant factor thereafter until the age of 15. Our findings challenged the established view that perseverance of effort (PE), rather than consistency of interest (CI), is the primary predictor of academic and sports performance^2,3^. In the current study, we expand and generalize these observations to a substantially different sport, alpine ski racing, and to a sample that includes athletes beyond the age range of the previous study (up to 19 years).

### Practice and grit in sports

The development of expertise in any domain hinges on acquiring domain-specific knowledge that enables individuals to recognize and respond to patterns and regularities within complex environments^4–6^. The role of practice is pivotal, as specialized knowledge is cultivated through sustained immersion in relevant activities^7^. Scientists have previously used retrospective methods to examine practice history profiles in sport and non-sport domains^8–13^. Findings have revealed a steady, year-by-year increase in practice as individuals develop, with a period of rapid growth during adolescence. More specifically, in our previous study on football players^1^ and alpine ski racers^9^ we found that around the age of 12, players began to engage in progressively greater amounts of domain-specific practice, leading to a curvilinear trajectory in practice accumulation.

Grit has increasingly attracted significant interest within the expertise literature^2^, particularly among scientists focusing on its relationship with practice behavior. Grit is defined as the persistent passion and unwavering determination to achieve long-term goals despite obstacles and setbacks^14,15^, and it is thought this personality trait can explain the intense and sometimes obsessive dedication to practice and competition seen in experts^16,17^. Positioned as a part of conscientiousness within the Big Five personality framework^18^, grit embodies the ability to persist in the face of challenges while working toward distant objectives^15,19^. Notably, grit differentiates itself from general conscientiousness through its stronger predictive power for success and its emphasis on sustained long-term effort over short-term intensity, which is more characteristic of conscientiousness^14,20^.

The construct of grit comprises two main components, namely, Perseverance of Effort (PE) and Consistency of Interests (CI). PE reflects an individual’s capacity to maintain effort and motivation when facing difficulties, while CI pertains to the sustained focus on a specific long-term goal without being diverted by new interests^21^. Tedesqui and Young^22^ describe CI as the direction of one’s passion and PE as the intensity of effort invested in pursuing that passion. Published reports highlight that grit is a strong predictor of success in academic settings, sometimes even surpassing the explanatory power of practice and innate ability^15,23–25^. A meta-analysis focusing on academic performance revealed that only PE significantly accounts for variance in outcomes and has a stronger criterion validity compared to CI^3^. Consequently, it is advised that researchers examine PE and CI separately rather than combining them into a single grit score^26^. In line with this recommendation, the present study treats the two components independently.

In the context of expertise, grit has also proven to be a valuable predictor of performance^2^. The influence of grit on expertise development is likely indirect, as personality traits generally impact factors related to expertise rather than expertise itself^27,28^. Practice is one such intermediary factor affected by personality traits like grit. Cormier et al.^2^ conducted a review identifying several studies that explored the relationship between grit and practice in both heterogeneous samples (comprising individuals with varying levels of expertise) and homogeneous samples (groups with similar expertise levels, such as all elite athletes or all amateurs). The general trend across these studies indicates that athletes exhibiting higher levels of grit tend to accumulate more practice hours and demonstrate greater persistence when encountering setbacks^22,27,29,30^, which is consistent with findings in non-sport domains^31^.

Cousins et al.^32^ found a significant correlation between the CI component of grit and the duration of athletes’ practice involvement, suggesting that sustained interest plays a role in prolonged engagement. This suggestion aligns with observations by Tedesqui and Young^22^, who reported that CI is more strongly associated with athletes’ decisions to continue or withdraw from sport activities. On the other hand, the research by Tedesqui and Young^22^ also indicated a stronger relationship between PE and the amount of practice undertaken. A subsequent study by the same authors^30^, however, did not replicate this specific relationship. These discrepancies highlight the need for further research clarifying these associations.

### Previous research on the association of grit with practice acquisition

Notably, no previous research has investigated the relationship between PE and CI grit components on practice acquisition, although we did demonstrate a positive association between total grit scores and individual practice hours in alpine ski racing^29^. Our previous research^1,27,28^ investigated the indirect relationship between grit (CE and PI components) and expertise in elite youth football players, specifically through the lens of accumulated practice. Although grit exhibited only a weak direct association with perceptual-cognitive abilities measured by objective tests, these abilities were significantly influenced by the amount of practice accumulated by age 14. The data revealed that practice hours were substantially affected by the players’ grit levels. Notably, the component of CI was positively correlated with the total accumulated practice, while PE did not show a significant link. These findings indicate that grit influences expertise indirectly by affecting how much the players have practiced up to that point in their careers.

In a subsequent study^1^, we aimed to uncover the mechanisms underlying grit’s impact on practice accumulation by utilizing retrospective estimates of practice hours. We found that elite youth football players maintained a steady amount of practice time during the sampling phase, up until the age of 12. The ages of 12 and 13 emerged as critical transition points, where players began to log increasingly more practice hours, continuing this upward trend until age 15. This escalation in practice was associated with grit; players with higher grit levels not only accumulated more practice in earlier years compared to their less gritty counterparts but also continued to increase their practice hours over time. What began as minimal differences eventually expanded into several hundred hours of additional practice by the early teenage years.

The accelerated increase in practice associated with grit was largely attributable to the consistency of interests component^1^. Players who demonstrated a sustained interest in football consistently invested more time in practice throughout their development. Furthermore, we discovered that PE had a modest yet significant effect on practice accumulation during the later specialization stage, from ages 12 to 15, when players intensified their practice routines. Although this effect emerged later and did not significantly influence the overall contribution of PE to total practice hours, it suggests that PE becomes a more relevant factor in the later stages of skill acquisition, as indicated by previous studies in both academic and sporting contexts e.g^3^.

### Current study

We broaden and generalize our previous findings on grit constructs and practice accumulation in youth football players by exploring a substantially different domain – alpine ski racing. The geographic, seasonal, and financial barriers to training in alpine ski racing raise important questions about how athletes accumulate practice hours and develop expertise under such constraints^33–35^. Alpine ski racing also offers a distinct context to investigate the role of psychological factors, such as grit, in sustaining long-term commitment and practice engagement despite limited opportunities and potential setbacks like injuries, which are prevalent in alpine ski racing considering it is a high-risk sport^36,37^. Critically, grit was not split into PE and CI components in our previous investigation^29^, although we did report a significant positive association between grit total scores and individual practice accumulation. Addressing assumptions regarding grit, practice accumulation, and expertise observed in other sports hold true in a domain like ski racing with its unique challenges has substantial scientific and applied value.

We utilize retrospective estimates of how much elite youth skiers from Austria and the USA have practiced starting from an early age – a method employed in several other studies^7^, including our own work on football players^1,27,28^ and ski racers^9,29,38^. However, the participants in this study are considerably older than previous work using football samples, as they are already in their late teenage years. This enabled us to go beyond age of 15 years, as in our previous study^1^, and explore later adolescent years. We employ a nonlinear modeling approach, Generalized Additive Models, GAM^39^ for our longitudinal data instead of traditional linear methods. The nonlinear flexibility allows us to capture the accelerated practice acquisition which we expect to find at around age 12 based on previous research^1,9^ without relying on the restrictive and potentially inaccurate assumptions inherent in quadratic or cubic functions^40,41^. GAMs can handle multiple nonlinear relationships simultaneously, allowing us to include other predictors of interest, such as grit or control variables like country and gender, each with its own smooth function. This approach facilitates a more nuanced analysis of how these factors influence practice trajectories.

We also differentiate between different kinds of practice in our estimates. Structured practice activities included competition, one-on-one coach-led practice, and group coach-led practice. Unstructured practice activities encompassed individual solo practice (i.e., practice apart from mandatory obligations), free play, and indirect activities such as playing ski-related video games or watching skiing on TV. While structured practice is closely related to the deliberate practice concept and an important predictor of expertise^10,42^, unstructured practice activities have also been shown to be indicators of future expertise due to their role in developing creativity and intrinsic motivation^43^.

Our study adopts a predominantly exploratory approach, yet the inclusion of a broader age range, a nonlinear analysis framework, and a detailed distinction between types of practice activities allows us to extend and generalize prior findings. For instance, we anticipate that elite youth alpine ski racers, like athletes in previous studies^8,10,12,13^ will begin to show an accelerated accumulation of practice around the age of 12. In contrast to most previous research, we provide formal analyzes for specifying the exact age when the acceleration of practice accumulation occurs.

Since our sample includes athletes beyond the age of 15, unlike our earlier study^1^, we also expect to observe a more nuanced differentiation in the association of grit’s subcomponents – consistency of interest (CI) and perseverance of effort (PE) – with accumulated practice, which was not available in previous ski racing datasets due to these subcomponents being summed as a total grit score^29^. Consistent with earlier findings^1,28^, we predict that CI will have a steady association with practice in the early years, up to around age 12. In contrast, PE may have limited association during this initial period but is likely to play a more critical role in later stages, as suggested by prior research in both sport^1,28^ and non-sport domains^3^. Finally, we hypothesize that unstructured practice activities are more strongly associated with grit than structured ones. Unlike structured practice, which is contingent on external factors such as coaching and organized training schedules, unstructured practice relies on the athlete’s own initiative, making it a more direct reflection of intrinsic motivation and commitment – qualities central to grit^14^. Within unstructured practice, we expect CI may be particularly associated with unstructured activities, as athletes with sustained interests are more likely to engage in self-driven practice and free play to deepen their connection to the sport. In contrast, PE might be more strongly associated with structured practice, particularly in helping athletes persist through demanding training sessions or competitive pressures.

## Results

### Descriptive results and (Non)Linearity check

**Fig. 1:**
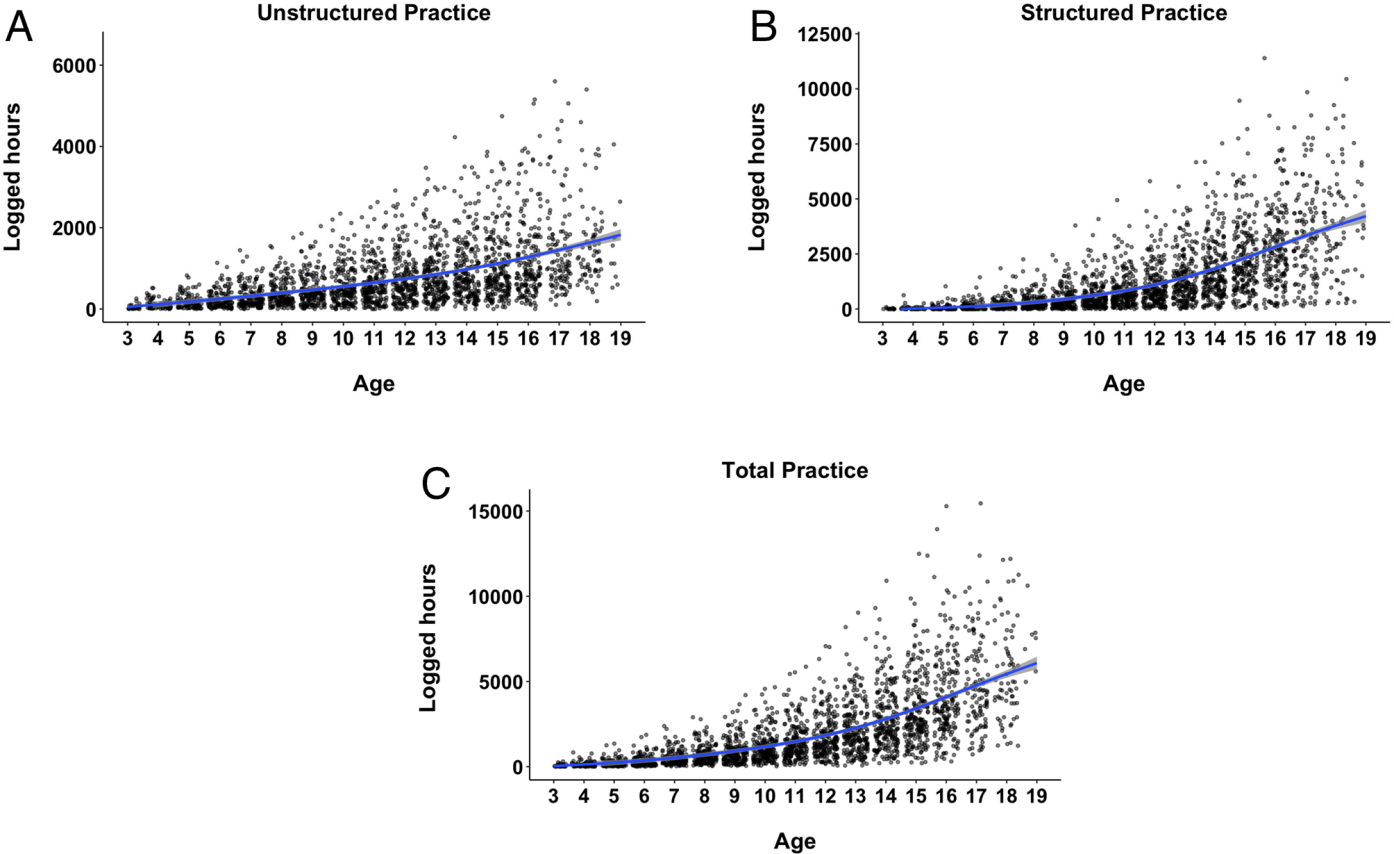
Accumulated Practice over Years. Dots represent individual data points, while the blue line represents the best non-linear (Logically Estimated Scatterplot Smoothing - LOESS) fit to the data.

Figure 1 shows the accumulated practice over time for all participants across unstructured practice activities (Fig. 1A), structured practice activities (Fig. 1B), and total practice (Fig. 1C). Skiers logged similar amounts of time each year until the age of 12 or so, after which practice time increased sharply. We tested for nonlinearity in the relationship between age and accumulated practice using GAMs. For each practice type, we compared a linear model (with age as a linear predictor) to a nonlinear model (with age as a smooth term). Across all practice types, the nonlinear GAM models fit the data significantly better than the linear models, confirming the presence of nonlinear trends. For the total practice, the nonlinear model explained more variance (R² = 0.49) than the linear model (R² = 0.44), indicating a highly significant improvement (F = 49, *p* <.001), with the significant smooth term for age capturing the U-shaped curve. The same was found for the structured practice (R² = 0.46 vs. R² = 0.41 for the nonlinear vs. linear – F = 49, *p* <.001). While the inflection point was less pronounced in the unstructured practice compared to the structured and total practice, the smooth term for age still captured subtle nonlinear trends (R² = 0.32 vs. R² = 0.29 for nonlinear vs. linear model – F = 23, *p* <.001). We also conducted the non-linear check for the individual types of practice activities – see Supplementary Material, SM, Sect. 1.

We next examined where this curvilinear trend begins, specifically identifying the point at which the acquisition acceleration is most pronounced (see Methods). For total and structured practice, the inflection point occurred around 12.6 years, whereas for unstructured practice, it was later, at approximately 14.5 years. This pattern suggests that the increase in practice time during this stage is driven by participation in organized activities, such as formal training and competitions, which leave less time for unstructured practice. The inflection point analysis for the individual type of practice activities can be found in SM (Sect. 1).

### Association between grit and accumulation of practice

We next conducted GAMs to examine cumulative practice over the years as a function of age and grit components – Consistency of Interest (CI) and Perseverance of Effort (PE) – in interaction with age. Different types of accumulated practice (e.g. unstructured, structured) served as the dependent variable, with age modelled non-linearly to capture its effect over time. Gender and Country were included as control variables. The inclusion of grit components, CI and PE, allowed us to assess their impact on practice. Interaction terms between age and these grit components were included to reveal how consistency and perseverance shape practice trajectories across the lifespan. Finally, we compared the new models with grit subcomponents to the previous one without grit – they should fit better if the grit is differently associated with practice accumulation at different ages.

**Fig. 2:**
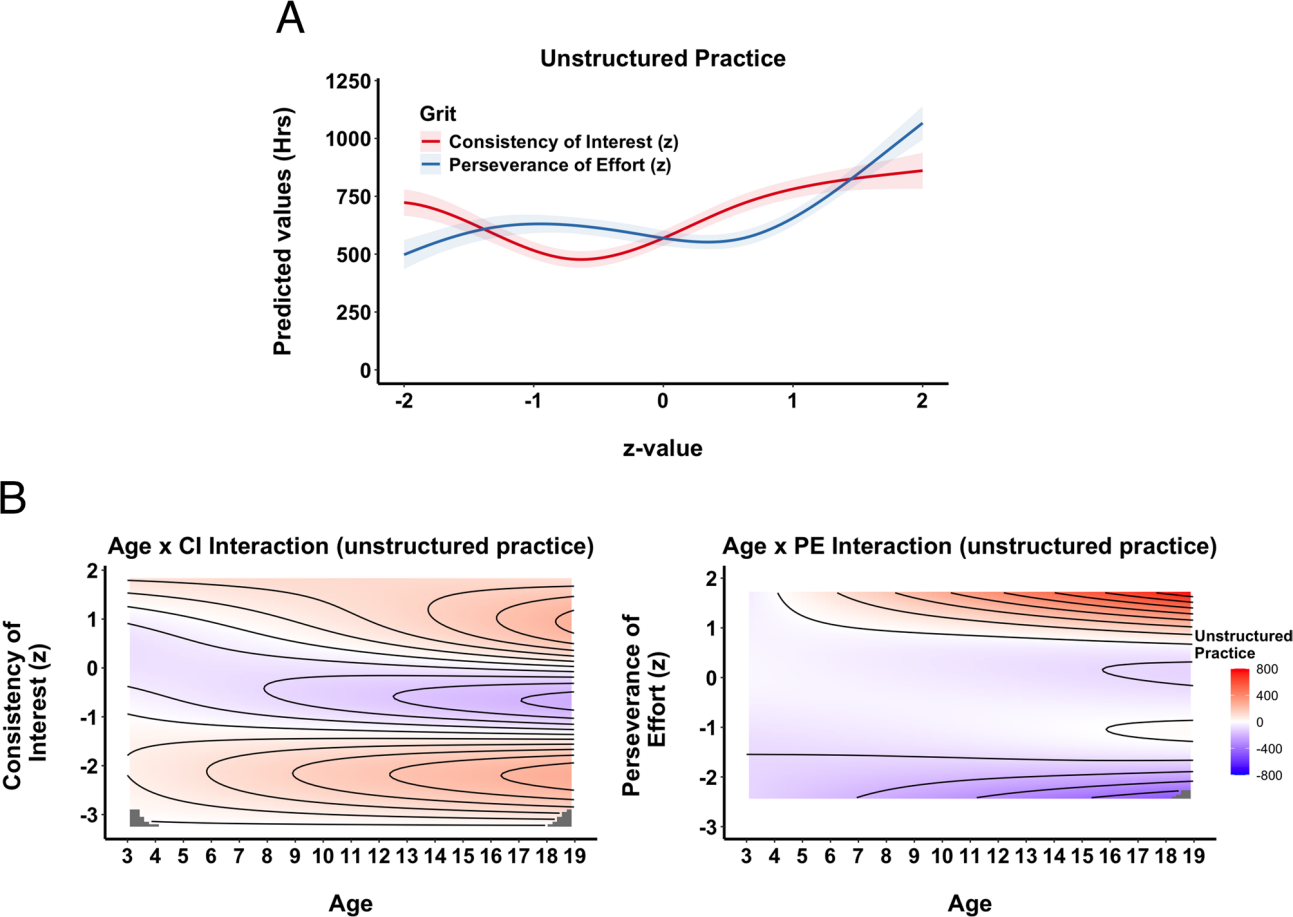
Unstructured Practice. (**A**) Partial Effects of Grit. Partial effect plots from the GAM illustrating the predicted hours of unstructured practice across standardized values (z-scores) of Consistency of Interest, CI, (red) and Perseverance of Effort, PE, (blue). Shaded areas denote ± 1 SE for the estimated mean. (**B**) Topographical Contour Plots of Interaction between Age and CI (left) and Age and PE (right). Each panel depicts the joint (interactive) effect of athletes’ age (horizontal axis) and one grit subcomponent (vertical axis) on the predicted hours of unstructured practice. The color gradient (blue to red) illustrates the degree of impact, and contour lines mark regions of similar partial effect, providing a “topographical” view of how CI and PE is associated with practice accumulation over time.

**Unstructured Practice**. The model with consistency of interest and perseverance of effort explained more variance than the model without them (R^2^ = 0.36 vs. R^2^ = 0.30) and fitted the data better (F = 14.9, *p* <.001). The GAM results demonstrate that both CI and PE do not have a significant linear relationship with accumulation of unstructured practice, but that the effect of each subscale varies over the developmental trajectory (for detailed GAM output, see SM, Sect. 2). To visualize how each subcomponent of grit is associated with unstructured practice, we generated partial effect plots based on our GAM. In these plots, we varied the z-scores for CI and PE individually from +/- two standard deviations from the mean, while holding all other predictors constant (e.g. setting the other grit subscale to 0). The y-axis displays the model-predicted total hours of unstructured practice, and the shaded bands represent ± 1 standard error around the estimated mean (Fig. 2A). Figure 2A shows that both CI and PE have a positive effect on the accumulation of unstructured practice only at the upper range (z-values: 0 to 1), but that there are no differences in contribution at the lower ranges (z-values: −2 and 0). In other words, differences in the CI and PE values matter for the accumulation of unstructured practice only in the higher CI and PE values.

This method, however, does not illustrate how the grit subcomponents are associated with accumulation across age (i.e. the interaction with age). We therefore provide topological contour plots in Fig. 2B which illustrate the joint (interactive) effects of age and each grit subcomponent on the accumulation of unstructured practice hours. The horizontal axis represents age, while the vertical axis displays standardized grit scores. The color scale (from blue to red) shows the partial effect on predicted practice, and the contour lines mark thresholds of similar effect levels. These plots allow us to see how the influence of each grit subcomponent on unstructured practice evolves over time. For instance, the Age × PE plot suggests that ski racers with higher levels of perseverance generally accumulate more hours as they get older, especially pronounced in the later teenage years. Meanwhile, the Age × CI plot reveals a subtler pattern: high CI values are linked to a modest increase in unstructured hours over age, though this effect varies by developmental stage. In this way, the contour maps complement the one-dimensional partial-effect plots (Fig. 2A), highlighting how age moderates the relationship between grit and unstructured practice.

**Fig. 3:**
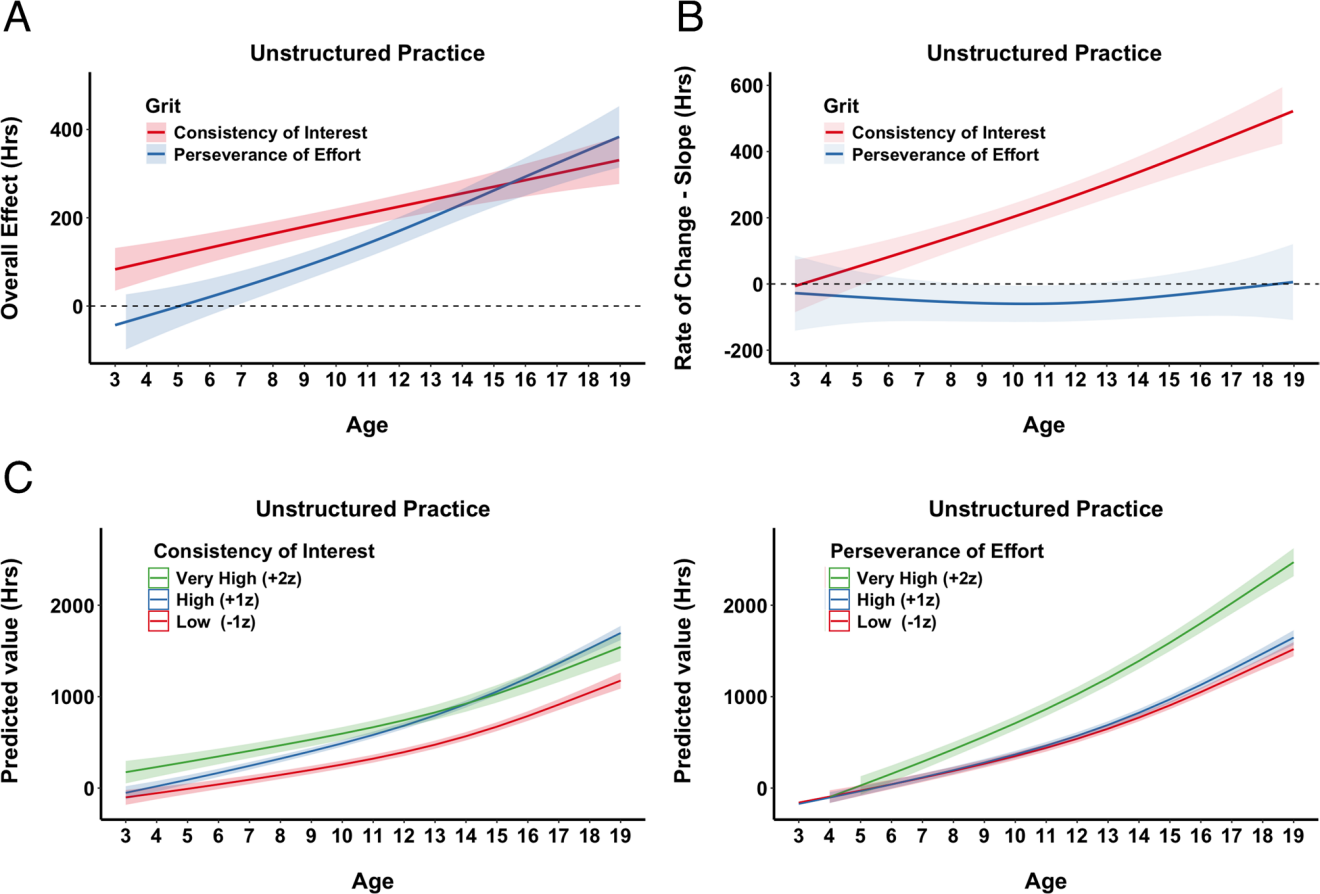
Unstructured Practice. **A**) Overall Effects of Grit Across Age. Illustrates the predicted overall impact of each grit subcomponent, CI (red) and PE (blue), on unstructured practice from ages 3 to 19. Estimates were computed by systematically varying age and holding other model terms constant, and then plotted the partial effect (in hours) with 1 SE confidence bands (shaded regions). The dashed horizontal line at 0 indicates no additional effect. **B**) The rate of change (slope) for CI and PE across age. Each line represents the estimated slope for a given grit subcomponent across ages 3 to 19. Higher (positive) slopes indicate that an incremental increase in the subcomponent accelerates unstructured practice hours at that age, whereas negative slopes imply the opposite. Shaded regions denote ± 1 SE around the estimated slopes. The dashed horizontal line at zero indicates the threshold where an effect changes from decelerating to accelerating practice accumulation. **C**) Hypothetical Accumulation Curves for Different Levels of Grit. Each panel depicts predicted unstructured practice hours from ages 3 to 19 for hypothetical skiers with low (–1 z), high (+ 1 z), or very high (+ 2 z) scores on CI (left) or PE (right). Shaded regions represent ± 1 SE around the estimates.

We visualize the cumulative impact of grit subcomponents on unstructured practice from early childhood through late adolescence by plotting the predicted overall effect of Consistency of Interest and Perseverance of Effort (Fig. 3A). Here, cumulative impact refers to the net association with each of the grit component across all ages examined, rather than focusing on a single age. In other words, these curves show how small differences in grit can build up over time, ultimately resulting in a greater divergence in unstructured practice hours by late adolescence. The curves indicate that both subcomponents exert an increasingly positive association on unstructured practice hours over time. Although the overall patterns appear similar, the effect of PE tends to rise slightly faster in later adolescence than does CI. These results reinforce that both components of grit become more influential as skiers progress in age, underscoring the growing importance of psychological traits for sustaining self-initiated practice.

To further explore how the impact of grit evolves over time, we estimated the rate of change (slope) of unstructured practice hours with respect to each grit subcomponent (Fig. 3B). Specifically, for every age from 3 to 19, we computed how small increases in CI or PE alter the slope (or acceleration) of the accumulation of the unstructured practice (Fig. 3B). A slope above zero indicates that higher grit values increase the pace at which practice accumulates; a slope below zero suggests they slow practice accumulation. Our findings show that CI exerts an increasingly positive rate of change over age, particularly from mid-adolescence onward. On the other hand, PE starts out with a slight negative slope in early childhood but transitions to a positive, and gradually increasing, effect by late adolescence. This finding suggests that CI becomes more critical for practice accumulation as ski racers mature, while PE maintains a consistent, baseline contribution throughout development.

Finally, we illustrate the GAM results by using hypothetical cases to show how grit components are associated with the accumulation of unstructured practice over time (Fig. 3C). We generated hypothetical scenarios representing low (–1 z), high (+ 1 z), and very high (+ 2 z) grit scores. For CI (Fig. 3C, left panel), the model suggests that adolescents who maintain a high (+ 1 z) and very high level of interest (+ 2 z) accumulate substantially more unstructured practice hours than those with low (–1 z) levels of interest, particularly during the mid- to late-teen years. Notably, the gap between high and very high CI narrows in later adolescence, implying diminishing returns for those who already score exceptionally high on interest. In contrast, PE (Fig. 3C, right panel) shows less separation among low and high across development. Yet, skiers with very high perseverance display a markedly steeper increase in unstructured hours toward the upper end of the age range. The PE becomes especially influential only at extremely elevated levels.

These graphical analyses of the GAM results reinforce that while both components of grit facilitate sustained self-initiated practice, CI steadily boosts practice accumulation across adolescence, whereas PE has more modest effects unless it reaches exceptionally high thresholds. This complements the findings from the interaction analyses, where CI and PE showed distinct and non-linear effects on practice trajectories across age. We further broke down the unstructured practice activities to self-training, play, and indirect activities and analyze them with GAMs (see SM, Sect. 3). As with the overall unstructured practice, in self-training and play activities we have situations where CI consistently drives practice accumulation, especially the curvilinear effect after age 12 when the skiers start accumulating more practice. On the other hand, PE’s influence is conditional on reaching very high levels and becomes even more impactful than CI towards the end of the period. The indirect activities are the only type of unstructured practice which does not follow the same pattern, as only PE has a positive impact.

**Fig. 4:**
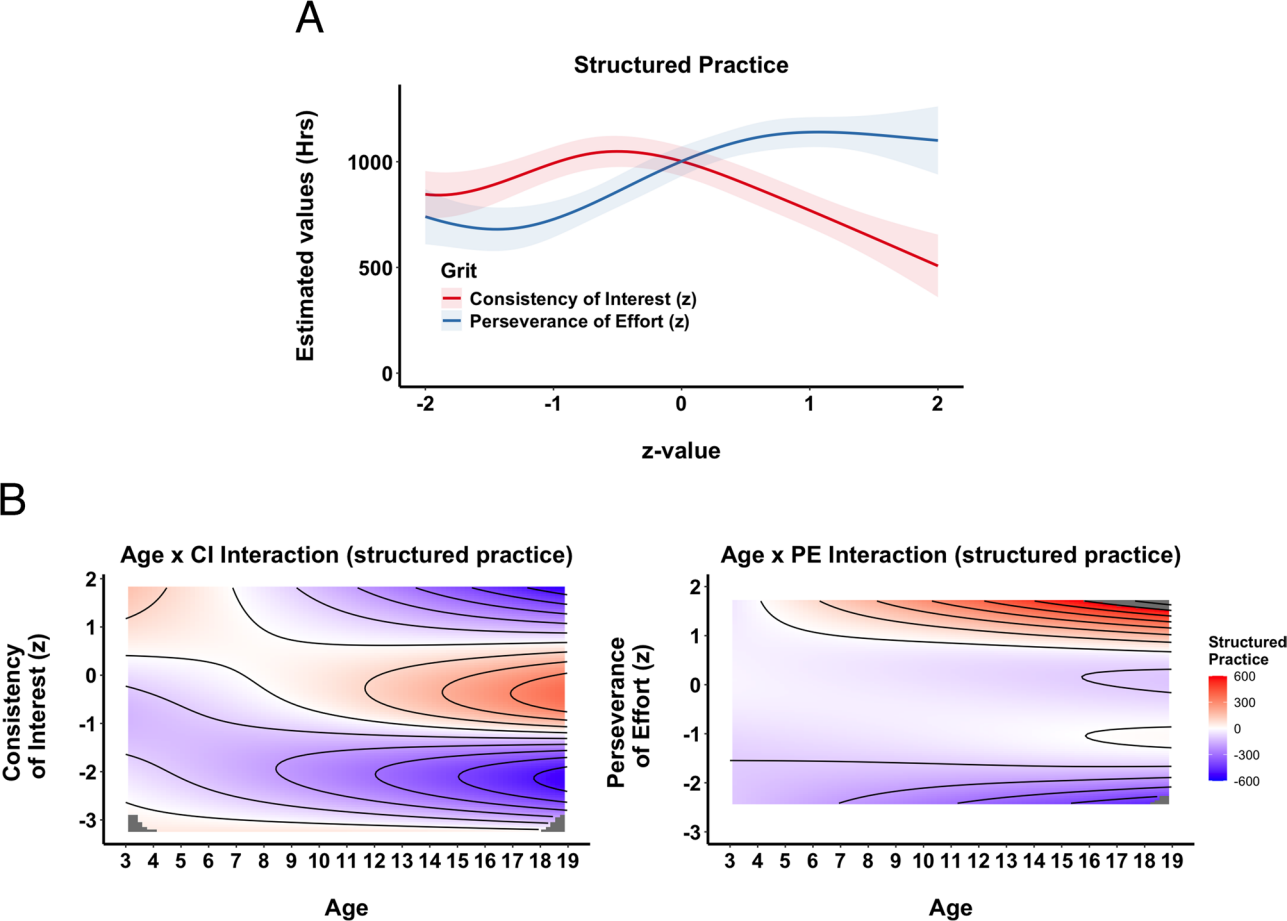
Structured Practice. **A**) Partial Effects of Grit. Partial effect plots from the GAM illustrating the predicted hours of structured practice across standardized values (z-scores) of Consistency of Interest, CI, (red) and Perseverance of Effort, PE, (blue). Shaded areas denote ± 1 SE for the estimated mean. **B**) Topographical Contour Plots of Interaction between Age and CI (left) and Age and PE (right). Each panel depicts the joint (interactive) effect of athletes’ age (horizontal axis) and one grit subcomponent (vertical axis) on the predicted hours of structured practice. The color gradient (blue to red) illustrates the degree of impact, and contour lines mark regions of similar partial effect, providing a “topographical” view of how CI and PE are associated with practice accumulation over time.

**Structured Practice.** At the age of our participants, structured practice, comprising official competitions and coach-led training (either individually or in groups), is largely beyond their direct control. Consequently, we did not expect grit’s subcomponents to have as pronounced an effect on the accumulation of these activities. Indeed, PE and CI explained slightly less variance here than in previous cases, although the model including grit’s subcomponents was still significantly better than the model without them (R^2^ = 0.49 vs. R^2^ = 0.47; F = 5.3, *p* <.001). The main effect of CI was not significant (see SM, Sect. 2, for detailed estimates and significance levels) and was even negative, indicating that structured practice accumulation decreased with higher CI scores (Fig. 4 A). By contrast, PE showed a significant positive impact, with structured practice steadily increasing alongside higher PE scores before leveling off at around + 1z (Fig. 4 A).

Both CI and PE significantly affected the accumulation of structured practice across age (interaction: age × CI/PE). As illustrated in Fig. 4B, CI had some influence early on, leading to higher amounts of structured practice for those with high CI scores. However, as skiers got older, high CI values eventually showed a negative impact. PE, on the other hand, had no effect very early on but increasingly contributed to structured practice accumulation for values above + 1z as participants aged.

**Fig. 5:**
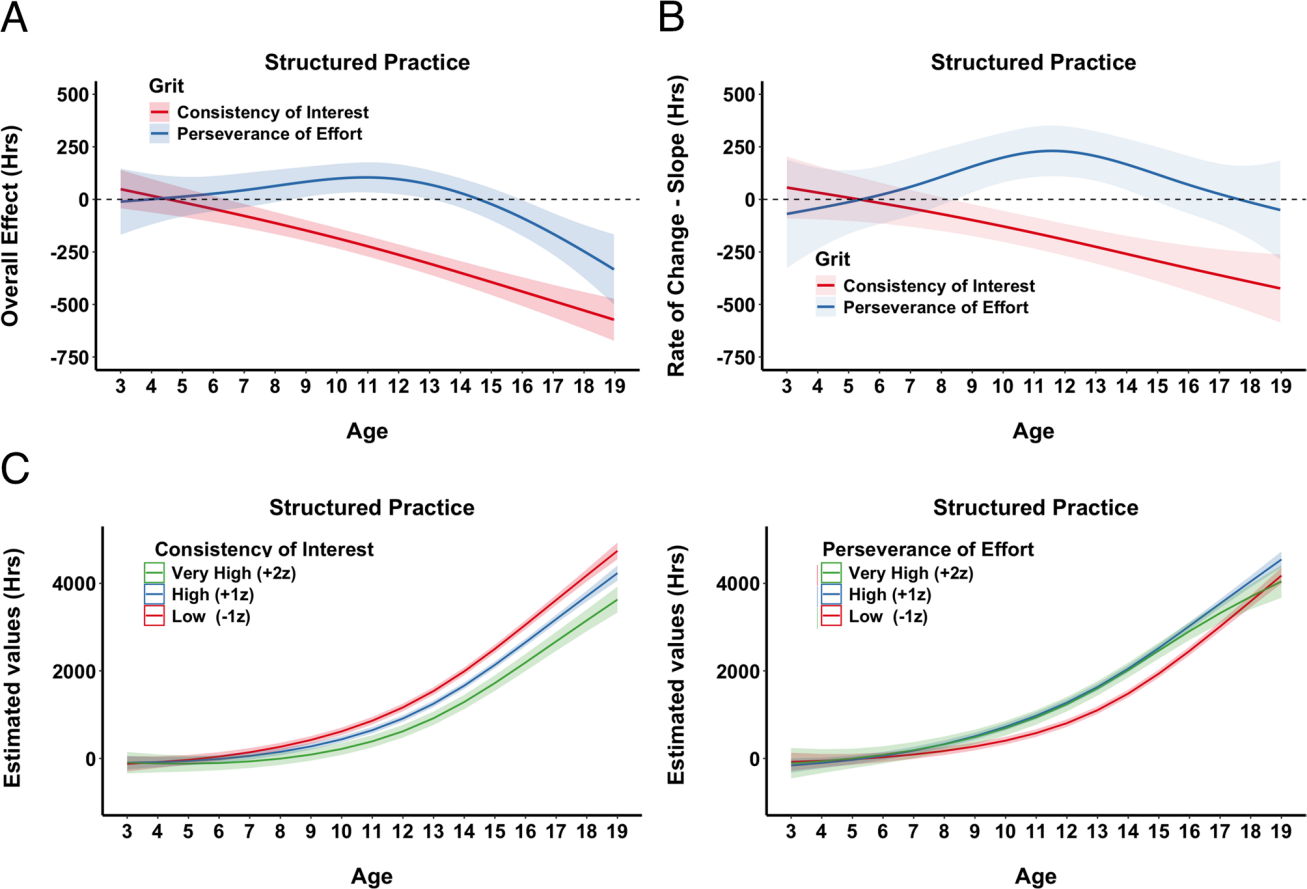
Structured Practice. **A**) Overall Effects of Grit Across Age. Illustrates the predicted overall impact of each grit subcomponent, CI (red) and PE (blue), on structured practice from ages 3 to 19. Estimates were computed by systematically varying age and holding other model terms constant, and then plotted the partial effect (in hours) with 1 SE confidence bands (shaded regions). The dashed horizontal line at 0 indicates no additional effect. **B**) The rate of change (slope) for CI and PE across age. Each line represents the estimated slope for a given grit subcomponent across ages 3 to 19. Higher (positive) slopes indicate that an incremental increase in the subcomponent accelerates structured practice hours at that age, whereas negative slopes imply the opposite. Shaded regions denote ± 1 SE around the estimated slopes. The dashed horizontal line at zero indicates the threshold where an effect changes from decelerating to accelerating practice accumulation. **C**) Hypothetical Accumulation Curves for Different Levels of Grit. Each panel depicts predicted unstructured practice hours from ages 3 to 19 for hypothetical skiers with low (–1 z), high (+ 1 z), or very high (+ 2 z) scores on CI (left) or PE (right). Shaded regions represent ± 1 SE around the estimates.

Figure 5A confirms that the overall effect of CI decreased with age, while PE remained relatively constant. Similarly, Fig. 5B shows that the rate of change (slope) for CI was negative as skiers grew older, indicating that the acceleration of structured practice accumulation was negatively impacted by CI. PE’s effect on the acceleration of accumulation also varied, starting with little to no impact early on, followed by a positive impact, and then no impact at later ages.

To illustrate how CI and PE shape structured practice activities over time, we plotted hypothetical cases (Fig. 5C). CI had minimal influence in early adolescence, with lower CI associated with greater structured practice. In contrast, higher PE values predicted more structured practice as skiers matured. We further analyzed individual structured activities such as competition and training with a coach, individual or group, with GAMs (SM, Sect. 3). Competition had a slight positive effect of CI at the very end, and no PE effect, while coaching, both individual and group, featured negative effects of CI and some positive effects of PE (for group coaching).

**Fig. 6:**
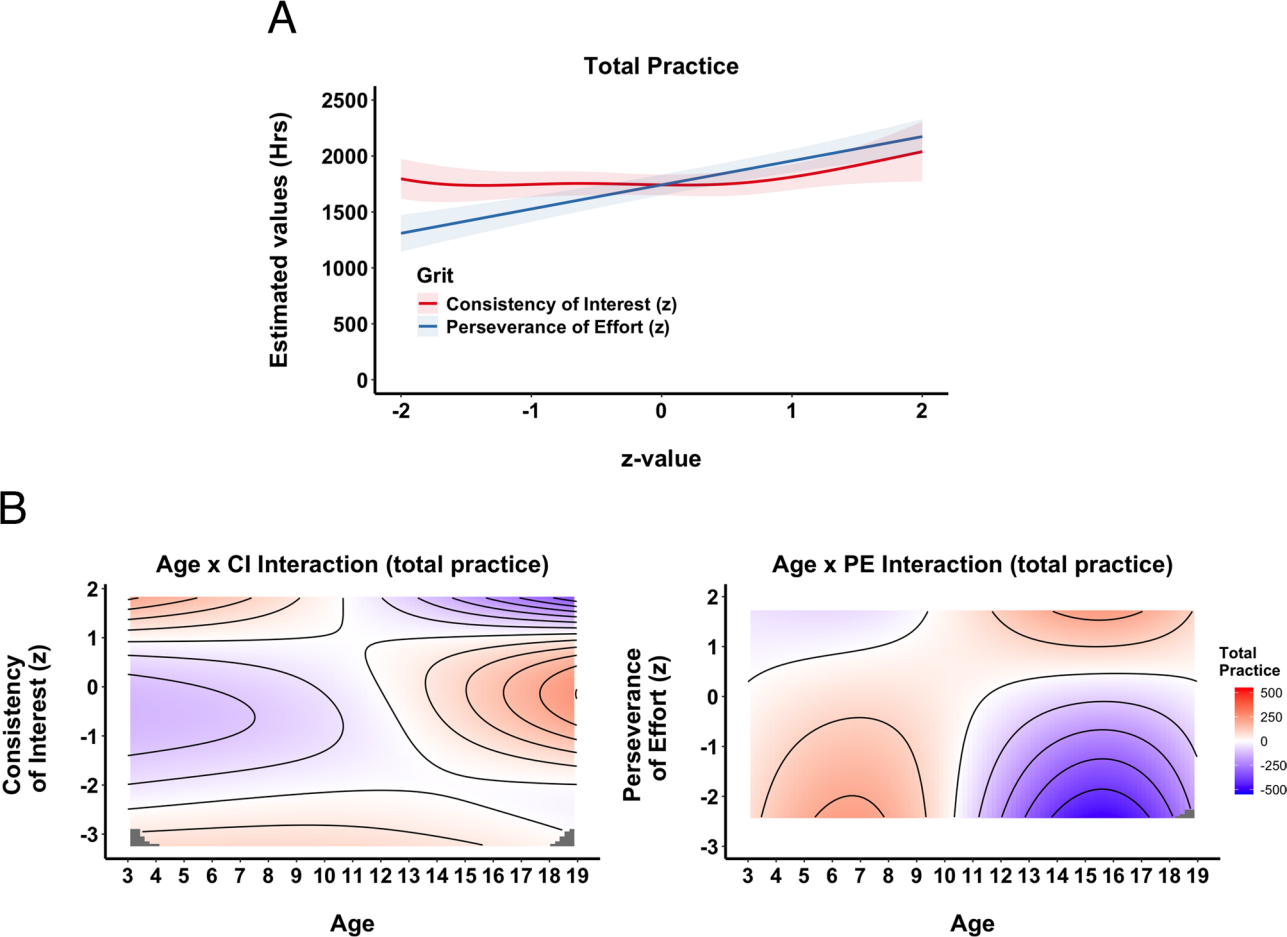
Total Practice. **A**) Partial Effects of Grit. Partial effect plots from the GAM illustrating the predicted hours of total practice across standardized values (z-scores) of Consistency of Interest, CI, (red) and Perseverance of Effort, PE, (blue). Shaded areas denote ± 1 SE for the estimated mean. **B**) Topographical Contour Plots of Interaction between Age and CI (left) and Age and PE (right). Each panel depicts the joint (interactive) effect of athletes’ age (horizontal axis) and one grit subcomponent (vertical axis) on the predicted hours of total practice. The color gradient (blue to red) illustrates the degree of impact, and contour lines mark regions of similar partial effect, providing a “topographical” view of how CI and PE are associated with practice accumulation over time.

**Total Practice.** We examined total accumulated practice by combining all structured and unstructured activities while excluding indirect activities. The GAM model explained total practice more effectively when both CI and PE were included (R^2^ = 0.52 vs. R^2^ = 0.50; F = 5.1, *p* <.001). The main effect of CI was not significant (see SM, Sect. 2), as there was little impact on the accumulated total practice irrespective of CI values (Fig. 6A). By contrast, PE showed a significant linear effect: skiers with higher PE scores accumulated more practice overall. Both CI and PE interacted significantly with age. As Fig. 6B illustrates, CI had a positive impact in the early years but diminished over time, eventually becoming negative. PE, on the other hand, showed minimal effect at younger ages but became more influential as athletes matured.

**Fig. 7:**
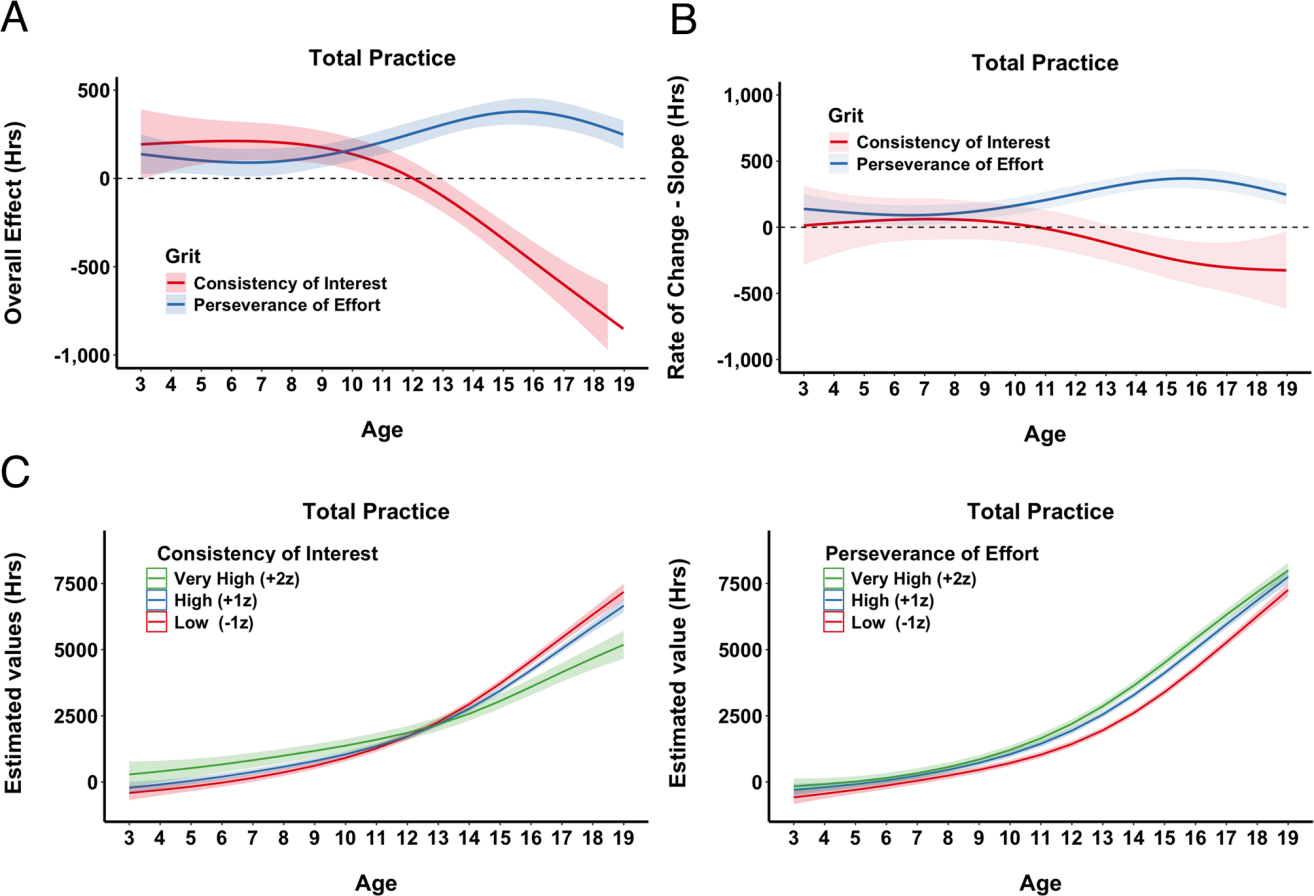
Total Practice. **A**) Overall Effects of Grit Across Age. Illustrates the predicted overall impact of each grit subcomponent, CI (red) and PE (blue), on total practice from ages 3 to 19. Estimates were computed by systematically varying age and holding other model terms constant, and then plotted the partial effect (in hours) with 1 SE confidence bands (shaded regions). The dashed horizontal line at 0 indicates no additional effect. **B**) The rate of change (slope) for CI and PE across age. Each line represents the estimated slope for a given grit subcomponent across ages 3 to 19. Higher (positive) slopes indicate that an incremental increase in the subcomponent accelerates total practice hours at that age, whereas negative slopes imply the opposite. Shaded regions denote ± 1 SE around the estimated slopes. The dashed horizontal line at zero indicates the threshold where an effect changes from decelerating to accelerating practice accumulation. **C**) Hypothetical Accumulation Curves for Different Levels of Grit. Each panel depicts predicted total practice hours from ages 3 to 19 for hypothetical skiers with low (–1 z), high (+ 1 z), or very high (+ 2 z) scores on CI (left) or PE (right). Shaded regions represent ± 1 SE around the estimates.

We explored this interaction further – Fig. 7A reveals that both CI and PE exerted similarly large effects early on; however, around age 10, CI’s association turned negative, whereas PE remained positive. The rate of change in total practice also differed between CI and PE (Fig. 7B): for the first 10 years or so, CI had little impact on the rate of change, but became slightly negative after age 12, whereas PE showed a positive slope later in adolescence. Finally, hypothetical cases (Fig. 7C) illustrate these patterns. Higher CI values promoted greater practice accumulation before age 12, but thereafter, skiers with lower CI scores tended to accumulate more total practice. Conversely, PE scores had no noticeable effect on acquisition rates until around age 12; from that point onward, athletes with higher PE scores steadily accumulated more overall practice.

## Discussion

Our primary aim was to explore how elite adolescent alpine ski racers accumulate both structured and unstructured practice over a broad developmental window, and to examine how the subcomponents of grit are associated with these trajectories. Based on previous research in youth sports, we expected that athletes would begin to show accelerated practice accumulation around age 12. Indeed, our findings confirmed that skiers log a consistent amount of practice during their early years until about age 12, after which they record a marked increase, especially in structured activities such as competitions and coach-led training. We also hypothesized that CI would be associated with accumulation of practice in the initial years, whereas PE’s association would become stronger in later adolescence, and this too aligned with our observations: CI maintained a positive effect on unstructured practice over time, whereas PE emerged as the driving force after age 12, accelerating the amount of self-initiated activities.

Additionally, we posited that unstructured practice, being more reliant on intrinsic motivation, would be more sensitive to grit than structured practice. Indeed, our data confirm that structured practice was considerably less associated with grit overall, with CI exhibiting either no main effect or a negative association, while PE displayed a positive but modest correlation that largely plateaued around + 1z. This divergence underscores how external constraints in structured contexts (e.g., mandatory competitions, coach-led sessions) can diminish or even invert the impact of high interest. In contrast, unstructured activities, where athletes exercise more autonomy, appear more strongly tied to both CI and PE across adolescence.

### Accelerated snowball effect

The increase in practice hours logged by elite players during early development, transitioning from steady growth to acceleration in adolescence, aligns well with previous research^9,44,45^. Our reported trends mirror those observed in our previous study with elite youth football players^1^ and other studies across men’s and women’s football competitions^46^. Around age 12, a pivotal shift occurs marked by athletes moving from parent-led participation to self-directed practice focused on sport-specific skill development^10,44,47^. This means that initial small differences in logged practice hours not only grow over time but become even more pronounced after age 12 – a phenomenon we previously labelled the accelerated snowball effect^1^.

Our findings confirm that structured activities demonstrated the greatest acceleration from childhood into adolescence, likely due to mandatory competitions and coach-led sessions. While unstructured practice also accelerates, it does so later and to a less pronounced degree. This discrepancy appears linked to the relative insignificance or even negative impact of CI in structured practice after a certain age, suggesting that external requirements overshadow intrinsic interest, or to the fact that PE only becomes influential in structured contexts when it is quite high (+ 1z or + 2z). By contrast, unstructured practice, though slower to accelerate, remains more responsive to both CI and PE, particularly in the mid-to-late teenage years, when self-directed motivation can play a larger role in practice engagement. This transition from fun-oriented engagement to focused practice is arguably one of the key predictors of expertise^10^.

### The role of grit in the acquisition of practice

One of the novelties of our study is that we differentiated practice activities instead of examining them collectively, as in our previous study^1^. This allowed us to gain deeper insight into how grit differently affects unstructured and structured practice activities. Unstructured activities, such as play, self-training, and indirect activities, are much more associated with individual traits than structured ones like competition and training with a coach. This distinction helps explain why grit’s subcomponents had a greater association with unstructured practice activities. CI exhibited a consistently positive impact throughout the years, while PE had a smaller effect early on but became more influential during childhood and adolescence. In contrast, only PE had a small impact on the acquisition rate of structured practice, whereas CI even showed a negative effect. One possibility is that elite youth skiers with especially high interest prefer more self-directed and playful activities, thereby reducing their relative involvement in externally scheduled or routine training sessions. Alternatively, they might find strictly managed environments insufficiently flexible or challenging, shifting more of their time toward unstructured pursuits that better align with their intrinsic motivation. As we documented in previous work^9^, many ski racers in the academy setting participate in other sports, and hours engaged in non-ski sports increase during adolescence which provides further opportunities for unstructured activity.

When structured and unstructured practice are combined as total practice (Fig. 6), we observe a pattern like our previous study with football players^1^. Specifically, CI drives the accumulation of practice early on but loses its impact around age 12, at which point PE takes over. This shift partly reflects the relative decline in CI’s association with structured practice by mid-adolescence, as well as the gradual drop in unstructured practice associated with high CI after age 12, thereby reducing its overall contribution to total hours. On the other hand, PE, which was initially less impactful, becomes more predictive from about age 12 onward, mirroring the transition toward more specialized training phases in youth sport. In other words, our total practice model essentially blends these opposing forces – CI’s diminishing effect on structured hours (and late drop in unstructured engagement) with its stronger association in the early years, plus PE’s growing impact in older adolescence – leading to the net pattern observed here and paralleling our earlier football findings.

Maintaining a steady interest in skiing during the initial stages appears to confer a positive effect, as athletes with consistent enthusiasm over time tend to engage more extensively in sport-specific activities, including both practice and play. This contributes to a higher total volume of involvement, particularly in the early years when most children are trying out multiple sports and distinctions between them are less pronounced. In contrast, the pronounced influence of perseverance of effort beyond age 12 among skiers expands upon observations from an earlier study of football players^1^, which only covered participants up to age 15. Our findings now indicate that the relatively modest effect of PE on practice accumulation, noted between ages 12 and 15 in younger football players, continues to grow and becomes more pronounced during later adolescence.

Our findings highlight that the two subcomponents of grit play distinct roles at different stages of a skier’s development. The PE component becomes increasingly crucial as athletes enter the later stages, particularly the investment phase after age 15. During this period, the complexity of training intensifies, and competition becomes more demanding, making it essential for athletes to persist through challenges and stay focused on their growth^48^. This trait, characterized by sustained commitment and vigorous pursuit of long-term goals^30^, enables young skiers to handle the escalating physical and mental demands of advanced practice and competition^13,49^.

In contrast, CI appears to be more influential during the early stages of sport engagement. At this point, athletes are still cultivating their motivation and commitment to continuous participation. A steady interest helps them remain engaged with sport-specific activities as they explore and develop their passion for skiing^30^. This aligns with the idea that consistency directs one’s passion toward specific goals, fostering early engagement and sustained involvement in the sport.

This pattern of results may go a long way to explaining the prevalence of connecting perseverance of effort with success in other sport-related studies^2,30^, as well as in a large meta-analysis in academic settings^3^. On the one hand, the kind of practice necessary for development of expertise is mostly acquired and exercised rather in teenage and late teenage years than in the years before. On the other hand, most participants in these studies were at least in late teenage years and their grit self-report may have been influenced by their realization about what is necessary for success, and it is certainly possible that different practice engagement trajectories have shaped personality characteristics like grit at the moment when data were collected retrospectively. This discrepancy when the participants were asked about grit might explain the absence of the effect of CI on subsequent success. Even in our study here, when the participants were relatively young at around 17 years old, their responses indicated that consistency of interest was only weakly related to the acquisition of practice in early years. That is in contrast with our previous study on elite 14-year-old football players, whose self-reports pegged CI seemingly more relevant factor in practice acquisition early on than it is the case here. Obviously, it is possible that differing sports may have something to do with somewhat different patterns of results, but the possibility that age at when responses are given to the grit questions is a factor should not be ruled out.

### Limitations

Unlike previous samples^1,28^, our sample is heterogenous in that it includes practitioners from different countries and gender. This heterogeneity likely avoids the problem of range restriction of observed effects^50,51^ in previous studies with homogenic samples. Nevertheless, as other studies on the similar topic, these findings are limited by several issues. First, retrospective self-reports of early activities, as used in this study, are prone to memory biases^52^ and could be cross validated with input from parents and coaches for greater accuracy^53^.

Second, personality traits such as grit exhibit moderate-to-high stability^54,55^. Meta-analytic evidence shows that rank-order stability for Big Five traits peaks around ages 16–18, with coefficients typically in the 0.60–0.80 range^56^. Specifically for grit, Duckworth and Quinn (2009) report a one-year test–retest correlation of *r* =.68 among adolescents^57^, and longitudinal studies spanning multiple waves report stability coefficients between 0.52 and 0.69 over three years^58^. In older or different populations, similar or even higher retest values have been observed – up to 0.84^59–61^. Moreover, given less-than-perfect temporal stability and measurement reliability, associations with pre-baseline practice are attenuated (e.g., √(0.60×0.80) ≈ 0.69). This would render our estimates conservative rather than inflated. Therefore, a single assessment of grit provides a proxy for earlier trait levels and is widely used in both retrospective and prospective research. Nevertheless, we acknowledge that grit can evolve over time in response to prolonged behavior, structured feedback, or athletic performance. For example, achievements and positive reinforcement in sport may further strengthen grit in more skilled athletes^62,63^.

While grit is assumed to influence practice, there is evidence that the relationship might be bidirectional. Grit could initially drive practice engagement, but success in practice might subsequently enhance grit, creating a feedback loop. This dynamic suggests grit may act along a trait-state continuum^28,31,64^, with consistent practice reinforcing personality traits like persistence. A longitudinal study design, incorporating annual measurements of grit and practice, could clarify these relationships^65^. Such an approach would also allow for supplementary data from parents, coaches, and other factors that contribute to skill acquisition. Because grit was assessed cross-sectionally, temporal precedence cannot be established.

## Conclusions

Even among talented young skiers, our findings suggest that individual differences in grit can lead to substantial variation in practice accumulation. Consistency of interest fosters initial engagement and sustained commitment during the early and mid-teen years, while perseverance of effort becomes the driving force that helps athletes navigate the heightened demands of specialized training and competition in later adolescence. Recognizing these distinct, developmental roles of grit’s subcomponents thus offers valuable insights into how athletes cultivate, and maintain, high levels of performance.

Ski racers with higher grit scores dedicate more hours to practice overall, and these small initial disparities can intensify over time, becoming especially pronounced in late adolescence. Although our cross-sectional approach to measuring grit and practice behaviors did not allow us to track changes in grit over multiple years, the results reinforce that grit can act as a key motivational mechanism underlying expertise development. Future research would benefit from longitudinal designs and repeated measures of grit, in order to capture potential shifts in both Consistency of Interest and Perseverance of Effort as athletes progress through increasingly demanding stages of development. This deeper understanding could inform targeted interventions or coaching strategies aimed at maximizing practice engagement, and ultimately, performance, in alpine ski racing and other sports.

## Methods

### Participants

Youth alpine ski racers were recruited from professional development ski boarding schools in Austria and the United States (*N* = 231, M_age_ = 16.7 ± 1.5, woman = 124) and athletes were categorized as “men” or “women” based on the events they competed in at the national and international levels. Ethical approval from the Institutional Review Board in the United States (University of Utah Sports Medicine and Sports Science Institute) was obtained. Prior to data collection, all participants provided written consent or assent (in addition to parental consent when applicable). All methods were performed in accordance with the relevant guidelines and regulations of the institutional review boards.

This study is part of a larger research project, so methods and data from this sample have been previously published^9,33,66–68^. However, the present study explores a new research question with distinct variables, analytical approaches, and theoretical contributions, including a unique introduction and discussion that sets it apart from earlier publications.

### Measures

**Practice.** To collect retrospective data on various aspects of each athlete’s engagement in alpine ski racing, including skiing milestones, time allocated to and involvement in other sports, a modified version of the *practice history questionnaire (PHQ)* was administered^69^. The skiing related milestones included the age at which participants commenced skiing; the age at which they first received supervision during skiing activities, the age at which they began physical conditioning, and the age of their inaugural competition. Furthermore, the questionnaire assessed different forms of engagement within ski racing, including the number of hours spent in competition, practice sessions led by a coach on a one-on-one basis, group practice sessions led by a coach, individual practice (i.e., practice sessions conducted independently, not obligatory), and free play. These engagement hours were further disaggregated into specific years, distinguishing between age categories such as U12 and U13. Additionally, the PHQ captured information on the duration of injury-related absences in each year, as well as the athlete’s participation in other sports throughout their lifetime. It should be pointed out, however, that the data were excluded from analysis, for a specific practice category, within a particular year, under two circumstances: firstly, if participants failed to report data for multiple months, and secondly, if they provided values exceeding the implausible number of hours per week (35 h) or year (2,000 h).

**Grit.** Perseverance and passion towards achieving long-term goals (grit) were evaluated using the *grit scale*^70^. The scale captured athletes’ current perceptions of their perseverance and consistency; no retrospective grit ratings were collected. In that sense, we treat grit as a rank-order stable trait. This scale comprised 12 items, with participants providing responses on a 5-point Likert scale ranging from “not like me at all” to “very much like me.” Previous research has demonstrated the grit scale’s robust internal consistency^70^, and in the present study, it exhibited good reliability (α = 0.78). We examined the two grit subcomponents separately, because it has been shown that they exhibit distinct associations with behavior and expertise-related outcomes^3,26^.

The scales captured current perseverance and consistency – we did not collect retrospective grit ratings. Measuring grit at earlier age was infeasible in this elite-athlete setting, and retrospective grit estimate would likely only add recall artefacts. We therefore follow common cohort practice of using a baseline trait snapshot with explicit non-causal interpretation.

### Procedure

In the United States, the data were collected from five distinct developmental ski academies located on the east and west coasts^9,29,66,71^. In Austria, data were collected from skiers attending boarding schools (specializing in skiing), that are situated in western and southern regions of the country. To ensure consistency, English versions of the questionnaires were utilized in both countries; however, the questionnaires were translated into German for the Austrian population by an experienced native German-speaker. Furthermore, the clarity of the translated questionnaires was examined by multiple expert German-English translators prior to their administration to Austrian athletes. In both countries, a dedicated workshop, lasting approximately 1–1.5 h, was organized by researchers and coaches to administer the questionnaire packages to the participating athletes. Clear instructions were provided to the participants, emphasizing the need to complete the packages on their own, independently, without seeking assistance from their peers or coaches. Although coaches were present and familiarized with the content of the packets, they were strictly prohibited from aiding athletes in completing the questionnaires.

### Data analysis

We employed Generalized Additive Models (GAMs) to examine the factors influencing accumulated practice hours. GAMs extend linear regression by allowing for non-linear relationships between predictors and the outcome variable through smooth functions^39,72,73^. One key advantage of GAMs is that they do not require the user to predefine the shape of the nonlinear regression curve; instead, the model determines it based on the data. This flexibility allows us to capture the accelerated practice acquisition during adolescence (from age 12 onward) without relying on the restrictive and potentially inaccurate assumptions inherent in quadratic or cubic functions^40,41^.

Moreover, GAMs offer several benefits over quadratic or cubic linear models in this context. They provide greater flexibility in modelling complex, nonlinear patterns in the data, enabling us to reflect the true nature of practice accumulation more accurately. GAMs can handle multiple nonlinear relationships simultaneously, allowing us to include other predictors of interest, such as grit or control variables like country and gender, each with its own smooth function. This approach facilitates a more nuanced analysis of how these factors are associated with practice trajectories. Additionally, GAMs improve interpretability by producing smooth functions that can be easily visualized and understood, illustrating how each predictor influences the response variable across its range. By avoiding the risks of overfitting or underfitting associated with specifying specific polynomial forms, GAMs provide a robust and flexible modelling framework suited to our longitudinal data.

Our primary GAM included accumulated practice of different types (total, structured, unstructured, or individual types of practice such as play, self-training, indirect, competitions, individual coach training, and group coach training) as the dependent variable. Predictor variables included Age (modelled as a smooth term to capture non-linear developmental effects), Gender, and Country (both included as control variables). To investigate the role of grit components, we added Consistency of Interest (CI) and Perseverance of Effort (PE) as linear terms to assess their main effects on unstructured practice. The interaction effects between age and the grit components were modelled using tensor product smooths (i.e. te(Age, CI) and te(Age, PE)), allowing us to examine how the impact of CI and PE varied across the lifespan. Importantly, grit is time-invariant in our data set; therefore each participant contributes one CI and one PE score that interact with the age-smooth term rather than analyses being run within separate age brackets.

The model fit was evaluated using adjusted R², deviance explained, and the REML (Restricted Maximum Likelihood) score, with higher values indicating better explanatory power. Hypothesis testing for smooth terms relied on approximate F-statistics and associated p-values, with significance indicating non-linear effects. Tensor smooths were interpreted using topological contour plots, which illustrate the interaction between age and grit components across the lifespan. Predicted values were generated for hypothetical cases to illustrate the overall effects of CI and PE at different z-scores (−1z, +1z, +2z) while holding other variables constant. Slopes were computed to quantify the rate of change in practice hours associated with CI and PE at different ages. Finally, the comparisons of predicted values across age provided insights into the relative contributions of CI and PE to practice accumulation.

We also used the flexibility of GAMs to identify the breakpoint – the age at which accumulated practice began to accelerate most sharply. By calculating the first derivative of the smooth function for age, we quantified the rate of practice accumulation over time. Subsequently, the second derivative, representing the change in the rate of accumulation (i.e., acceleration), was computed to pinpoint the age of maximum acceleration. This method allowed us to objectively determine the inflection point, capturing the transition to rapid practice growth during adolescence. To corroborate the results of the GAM-based breakpoint analysis, we employed segmented regression^74^ using the segmented package in R^75^. A piecewise linear regression model was fitted with an initial guess for the breakpoint at age 12, allowing the model to estimate the exact inflection point. The breakpoint estimated by the segmented model closely aligned with that identified by the GAM-based approach, supporting the robustness of the findings.

## Supplementary Information

Below is the link to the electronic supplementary material.

Supplementary material 1 (DOCX 7911.9 kb)

## Data Availability

The data and the code used for the analyzes can be retrieved from https://osf.io/za6v9/?view_only=2b0736a3384c4d1e98cb5b47abe12831.
