# Supplementary material for: Grit subcomponents are differentially associated with practice trajectories underlying expertise development
Source: Sci Rep. 2025 Oct 29;15:37812. doi: 10.1038/s41598-025-22533-x (PMC12572304; doi:10.1038/s41598-025-22533-x)
Supplement: Supplementary file 1 — Supplementary material 1 (DOCX 7911.9 kb) [file 41598_2025_22533_MOESM1_ESM.docx]

**Supplementary Material for:**

**Grit Subcomponents Differentially Predict Practice Trajectories**

**Underlying Expertise Development in Alpine Ski Racing**

Dijana Cocić^1^ Brady S. DeCouto^2^ Bradley J. Fawver^3^ Rhiannon L. Cowan^4^

David T. Hendry^1^ A. Mark Williams^5^**^†^** Merim Bilalić^1*^**^†^**

1 Northumbria University at Newcastle, UK

2 Florida State University, Tallahassee, Florida, USA

3 Walter Reed Army Institute of Research – West, Joint Base Lewis-McChord, Washington, USA

4 University of Utah, Salt Lake City, UT 84132, USA

5 Loughborough University, UK

***Corresponding Author**:

Merim Bilalić, Northumbria University, School of Psychology, Ellison Square, NE1 8ST Newcastle, UK, +44 191 227 3291, [merim.bilalic@northumbria.ac.uk](mailto:merim.bilalic@northumbria.ac.uk)

**^†^ These authors share the senior authorship.**

**This PDF file includes:**

Supplementary text

Table SM1

Figures SM1 to SM7

**Other (online) supplementary materials (oSM) for this manuscript include the following:**

<https://osf.io/za6v9/?view_only=2b0736a3384c4d1e98cb5b47abe12831>

**Section 1. Individual Type of Practice – Descriptives and inflection point analysis**

In the next step, we checked the individual activities (Figure SM1). Skiers increased all individual practice activities but the increase is particularly visible in the structured activities such as coaching, individually or in group, and competition. The formal analysis confirms that the models with the non-linear fit were better than the models with the linear fit for the structured activities. For the Competition activity, the nonlinear model explained more variance (R² = 0.29) than the linear model (R² = 0.26), indicating a highly significant improvement (F = 26.44, *p* < .001), with the significant smooth term for age capturing the nonlinear relationship. The same was found for the Coach group activity (R² = 0.31 vs. R² = 0.28 for the nonlinear vs. linear – F = 21.04, *p* < .001). For the Individual training with coach, the nonlinear model also explained more variance (R² = 0.20 vs. R² = 0.16 for the nonlinear vs. linear model), with a significant improvement in model fit (F = 27.29, *p* < .001), and the smooth term for age capturing subtle nonlinear patterns.

While the increase may have been less pronounced in the unstructured activities, it was enough for the nonlinear models to prove better fitting than the linear ones in most activities. For the Self-training activity, the nonlinear model explained more variance (R² = 0.17) than the linear model (R² = 0.15), indicating a significant improvement (F = 20.49, *p* < .001), with the smooth term for age (p<.001) capturing the nonlinear trend. A similar result was found for Indirect activities; the nonlinear model performed better (R² = 0.15 vs. R² = 0.13 for nonlinear vs. linear – F = 17.93, *p* < .001). However, the Play activity did not display the same curvilinear pattern as the improvements were steady across the whole age range (R² = 0.20 vs. R² = 0.20 for nonlinear vs. linear – F = 1.27, *p* = 0.22).


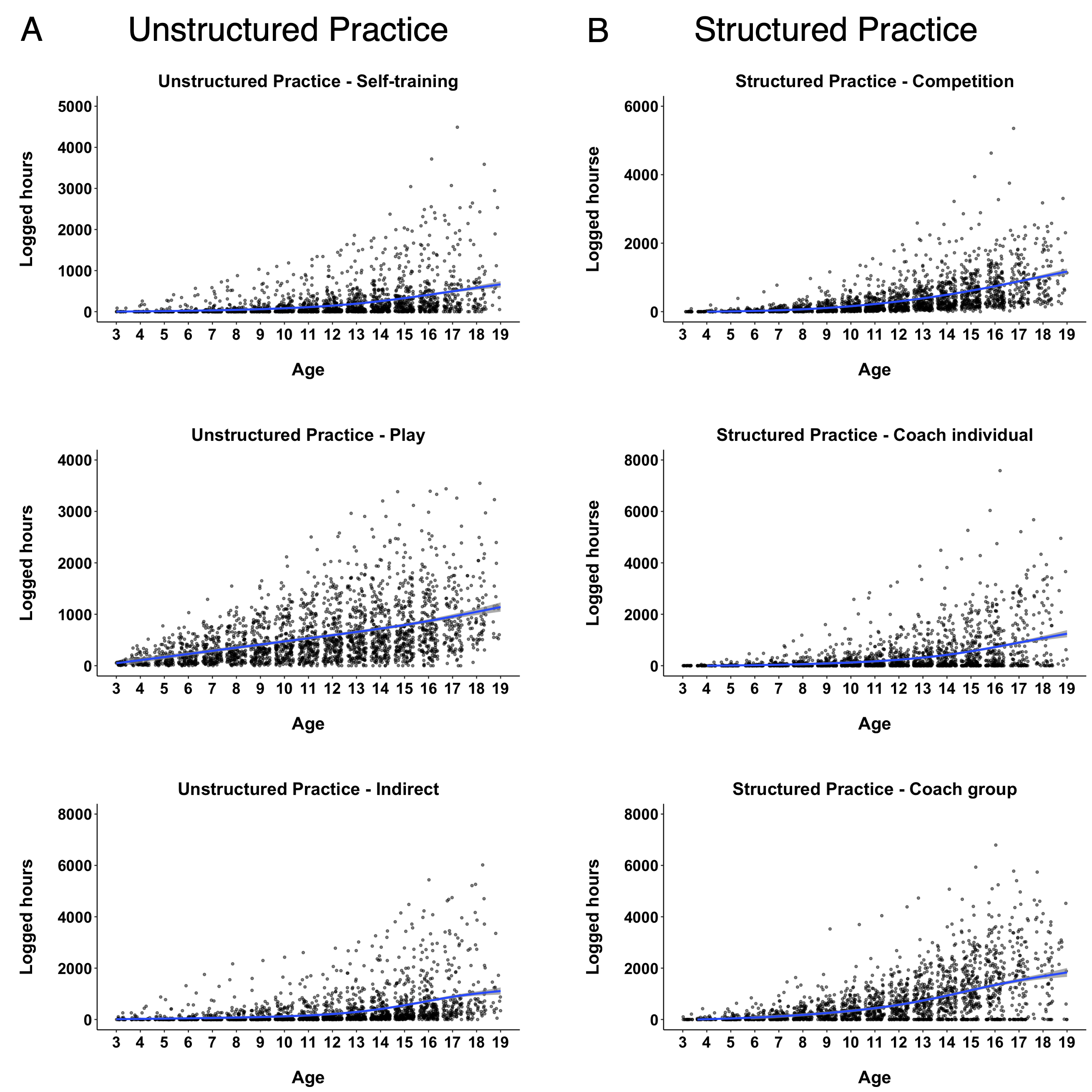


**Figure SM1. Different Types of Practice Accumulated over Years.** A) Unstructured practice (self-training, play, and indirect activities. B) Structured practice (competition, individual training with coach, group training with coach). Dots represent individual data points, while the blue line represents the best non-linear (loess) fit to the data.

Section 2. GAM Output for main model

Table SM1. GAM Output for Unstructured, Structured and Total Practice Activities

| **Parameters** | **Unstructured** | **Structured** | **Total** |
| --- | --- | --- | --- |
| **(Intercept)** | 592.53 (25.68)*** | 1141.65 (48.66)*** | 1740.39 (60.89)*** |
| **Gender1** | 81.71 (25.66)** | 36.76 (47.10) | 117.08 (61.39) |
| **CountryUSA** | 169.51 (28.63)*** | 111.35 (52.68)* | 277.88 (68.87)*** |
| **CIz** | 46.62 (29.60) | -98.58 (55.29) | -5.79 (68.36) |
| **PEz** | -25.59 (35.67) | 234.21 (102.69)* | 156.04 (67.75)* |
| **s(Age)** | edf=2.918 (F=18.327)*** | edf=4.203 (F=46.386)*** | edf=4.272 (F=49.060)*** |
| **te(Age, CIz)** | edf=4.433 (F=2.911)*** | edf=4.404 (F=1.698)*** | edf=3.027 (F=0.359)* |
| **te(Age, PEz)** | edf=5.172 (F=3.439)*** | edf=8.218 (F=1.921)*** | edf=1.924 (F=0.524)*** |

**p* < 0.05, ***p* < 0.01 ****p* < 0.001

Section 3. GAM Analyses for Individual Types of Practice

**Unstructured Practice – Self-training.** We used the same approach for the specific activities types on their own. First, we checked the accumulated self-training. The model with the CI and PE in it explained more variance and was a significantly better fit to the data than the model without CI and PE (R^2^ = 0.24 vs. R^2^ = 0.18; F = 14.3, *p* < .001). Figure 2 and Figure 3 graphically present the results of this model in the same manner for the unstructured practice. Figure SM2A shows the overall effects of CI and PE on the accumulated self-training – whereas there was a small increase in impact of CI with higher values (> 0z) compared to those with lower (< 0z), there was a big impact on accumulation of self-training for higher PE skiers (> +1z). The topographical graphs of the interaction between age and grit’s subcomponent (Figure SM2B) confirm this observation, but also provide additional information that the influence of high values of PE on self-training is particularly strong at a later stage starting from age 12. The graph for the overall effects across age (Figure SM2C) confirms that the PE becomes a stronger influence than CI after age 12. However, the rate of change in accumulation of self-training graph (Figure SM2D) indicates that CI is gaining impact as skiers age.

We also present the hypothetical cases in Figure SM2E. PE has generally higher impact on self-training than CI, confirming the overall effect analysis. However, CI impacts the rate of self-training acquisition beyond age 12 more than PE, as indicated in the rate of change graph (Figure SM2D). The difference between skiers who score high (+1z) and low (-1z) on CI is more pronounced than the for the same values in PE where they produce no differences in the self-training accumulation rates. However, once we add individuals with very high scores (+2z), the PE becomes highly impactful whereas CI does not change much from the previous values.

As with the overall unstructured practice, we have a situation where CI consistently drives self-training accumulation, especially the curvilinear effect after age 12 when the skiers start accumulating more practice. On the other hand, PE's influence is conditional on reaching very high levels and becomes even more impactful than CI towards the end of the period.


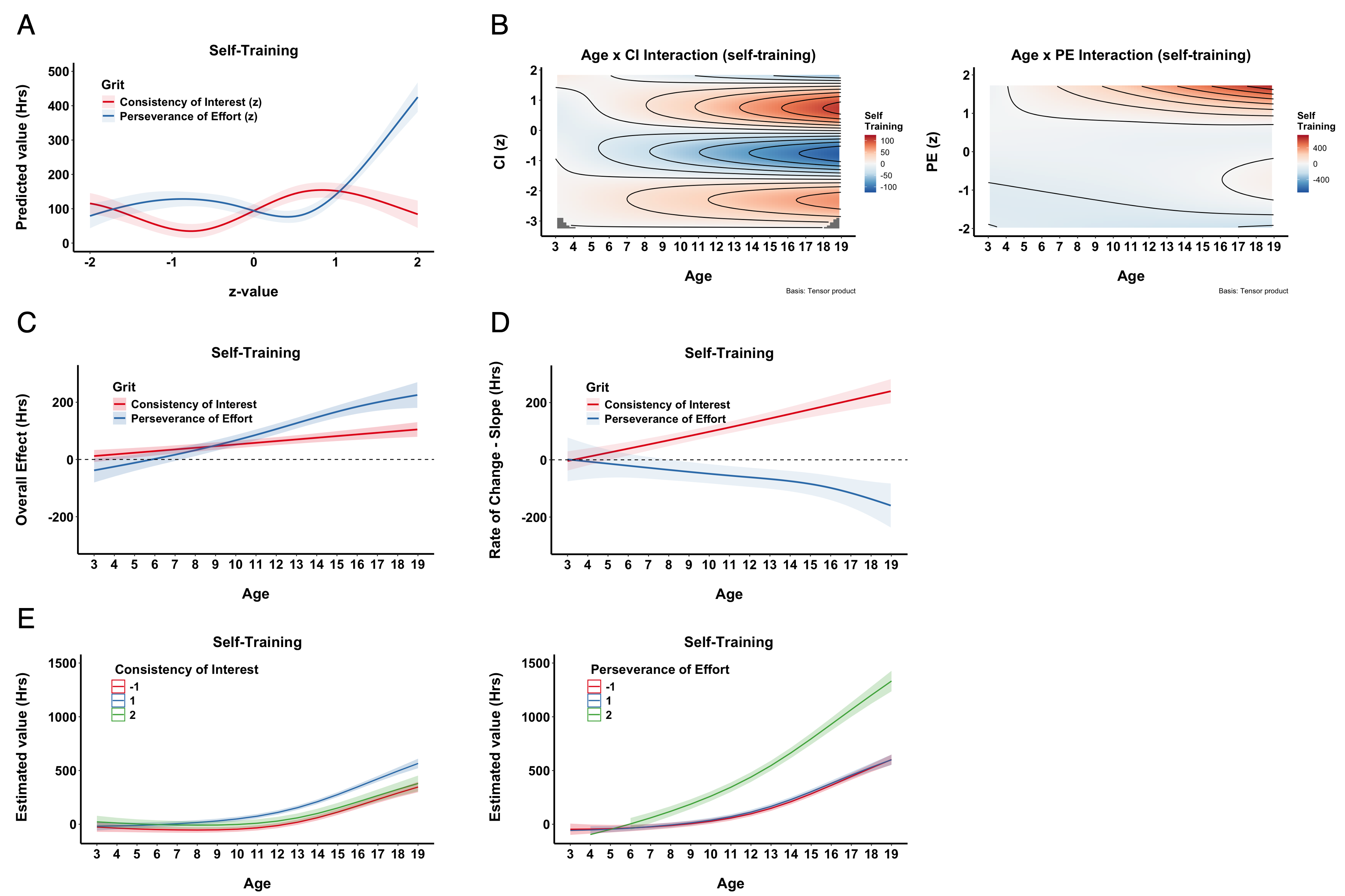


**Figure SM2. Self-Training (Unstructured Practice).** A) Predicted values across values of consistency of interest (CI) and perseverance of effort (PE). B) Interaction between age and CI (left) and PE (right) across age. C) Overall effects of CI and PE across age. D) The rate of change for CI and PE across age. E) Hypothetical practice curves over age for very high (+2z), high (+1z) and low (-1z) values for CI and PE. Dashed lines in C and D represent 0 or no effect. Shaded areas around the mean are +/- 1SE.

**Unstructured Practice – Play.** The play activities were also improved by adding CI and PE in the model (R^2^ = 0.27 vs. R^2^ = 0.24; F = 9, *p* < .001). Both CI and PE had a positive impact on the accumulation of play activities across their range of values (Figure SM3A). The impact of both subcomponents was increasing with the higher values, particularly for high values of CI. The interaction graphs (Figure SM3B) indicate that the impact of both CI and PE increases with age, while the graphs with the overall effect across age (Figure SM3C) confirms that this is the case. The CI has generally a stronger overall influence on play than PE does, part of which is due to its continually rising impact on the acquisition of play across age (rate of change – Figure SM3D).

The hypothetical cases in Figure SM3E confirm that the overall CI has more impact than PE, and that the impact of high vs. low among CI values has a particularly strong effect on play acquisition at later stages. As in the previous analyses, adding a particularly high value (+2z) does not impact the CI influence on play, but it has a pronounced effect of PE’s impact, which suddenly becomes pronounced.


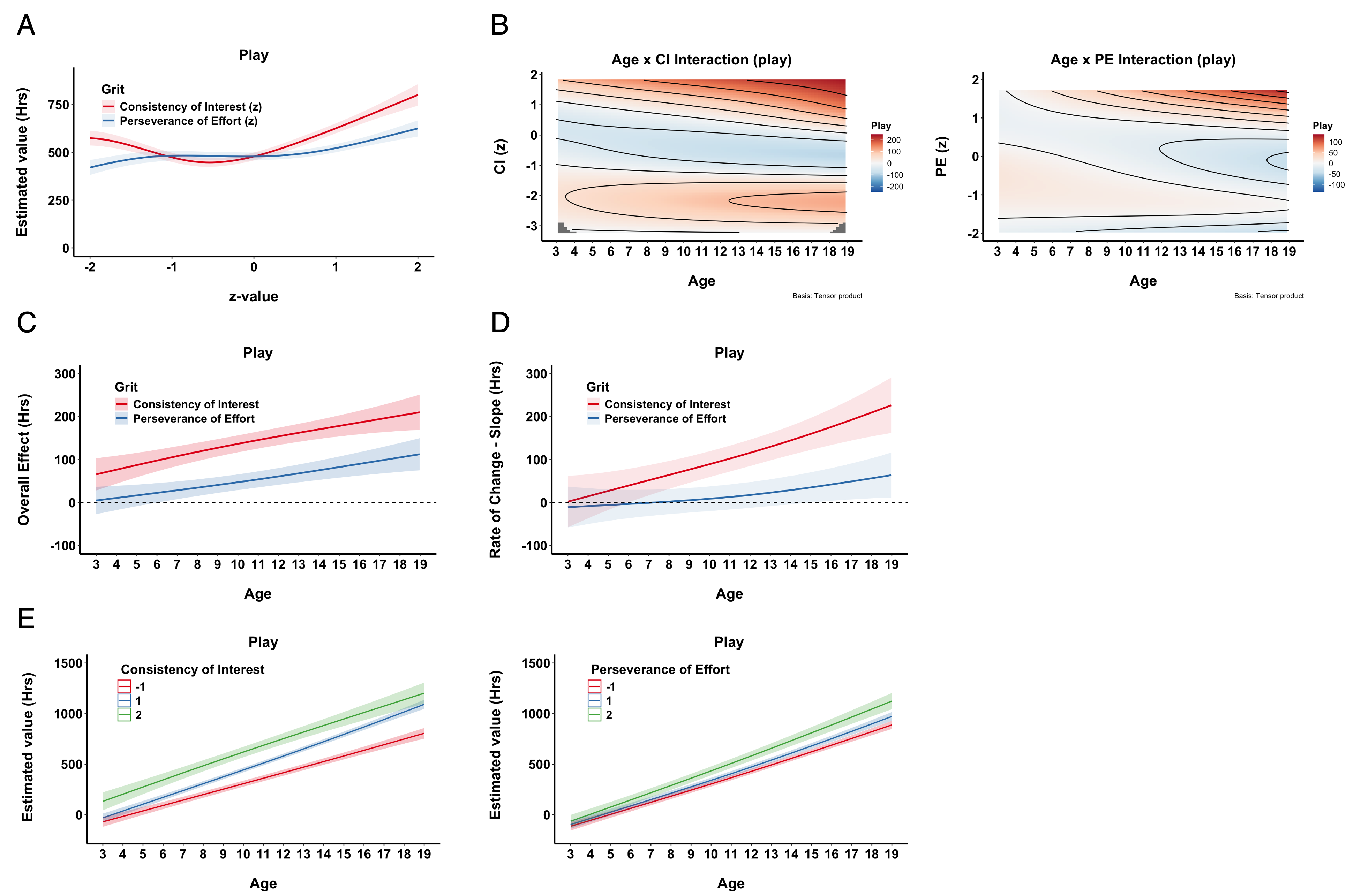


**Figure SM3. Play Activities (Unstructured Practice).** A) Predicted values across values of consistency of interest (CI) and perseverance of effort (PE). B) Interaction between age and CI (left) and PE (right) across age. C) Overall effects of CI and PE across age. D) The rate of change for CI and PE across age. E) Hypothetical practice curves over age for very high (+2z), high (+1z) and low (-1z) values for CI and PE. Dashed lines in C and D represent 0 or no effect. Shaded areas around the mean are +/- 1SE.

**Unstructured Practice – Indirect activities.** Finally, we checked the indirect activities which we initially did not include in the unstructured practice due to their non-participatory nature. The CI and PE contributed to a significantly better model (R^2^ = 0.24 vs. R^2^ = 0.20; F = 6.5, *p* < .001), with both CI and PE positively influencing the time spent on indirect activities (Figure SM4A). The impact of both CI and PE was bigger as the scores on CI and PE were higher. The influence over the age (Figure SM4B) is different, with high values of CI having an impact on indirect activities at early stages but slowly fading towards the end of the period. In contrast, PE has no such early influence, but its high values do impact the acquisition of indirect activities at the later ages. The overall effect across age graph (Figure SM4C) confirms that the PE has a small but ever-increasing influence on the indirect activities over the years. However, the CI influence becomes negative with age. The same situation is found when we look at how the CI and PE impact the acceleration of indirect activities over years – PE has almost no discernible effect except towards the end of the developmental period, while the CI’s influence becomes profoundly negative with age (Figure SM4D).

The hypothetical cases (Figure SM4E) demonstrate that the PE has overall more impact on the indirect activities across age, particularly at the later stages. PE also somewhat differentiates between high and low values, as skiers with very high PE values (+2z) spend more time on indirect activities than their peers with lower PE values (+1 and -1). No such pattern was found in CI, where the low values had even more positive impact on the indirect activities than high and very high values.


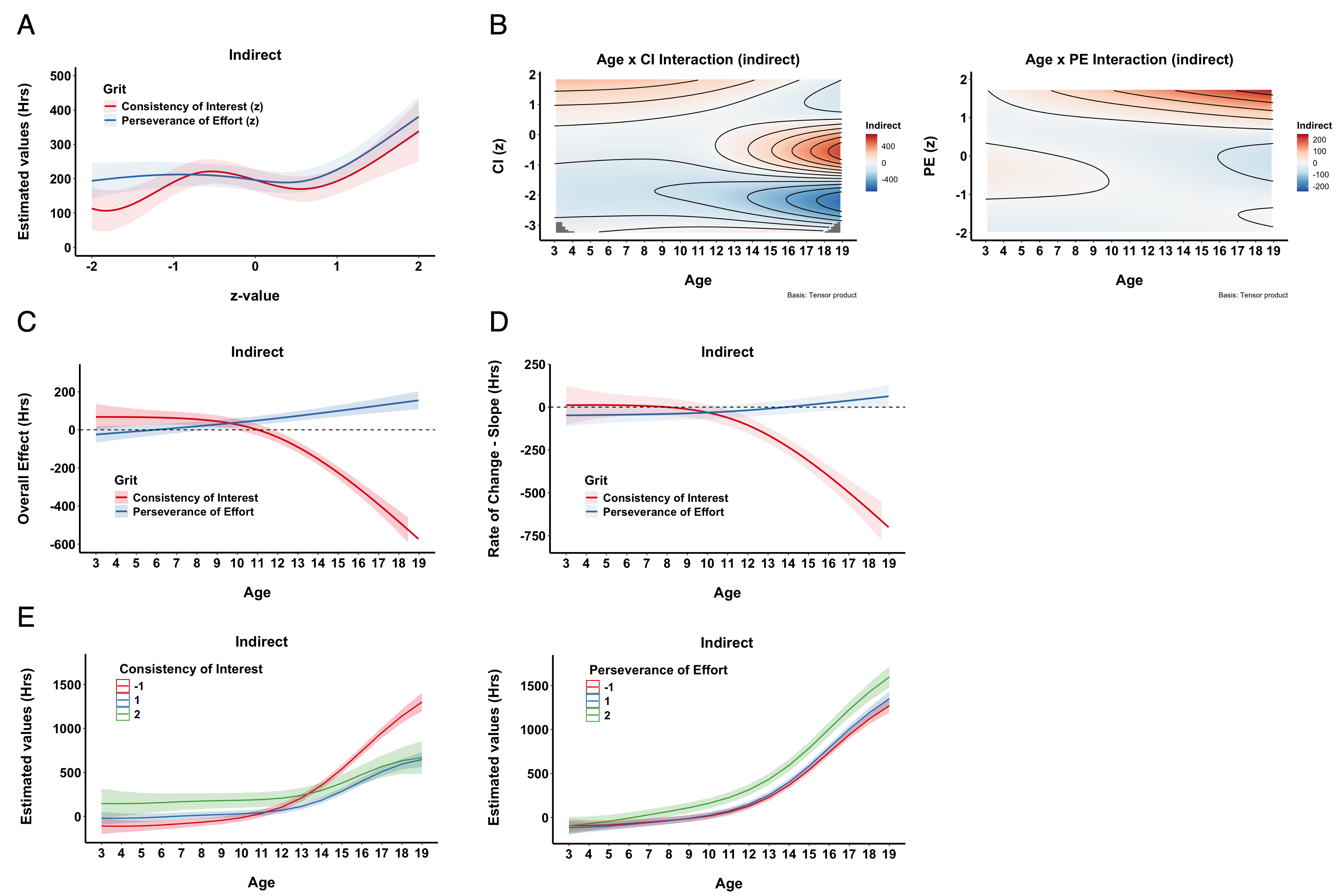


Figure SM4. Indirect Activities (Unstructured Practice). A) Predicted values across values of consistency of interest (CI) and perseverance of effort (PE). B) Interaction between age and CI (left) and PE (right) across age. C) Overall effects of CI and PE across age. D) The rate of change for CI and PE across age. E) Hypothetical practice curves over age for very high (+2z), high (+1z) and low (-1z) values for CI and PE. Dashed lines in C and D represent 0 or no effect. Shaded areas around the mean are +/- 1SE.

**Structured Practice – Competition.** We next looked at individual activities within the overall structure practice. Although the difference was not pronounced as in the unstructured practice activities, CI and PE added some value to the model of predicting the competition (R^2^ = 0.38 vs. R^2^ = 0.37; F = 4.1, *p* < .001). There was hardly any effect of CI and PE on the accumulation of competition practice, with a slight exception for high values of CI (Figure SM5A). The increase of impact for higher values of CI is particularly visible at later years, whereas PE continues to exert little influence over the years (Figure SM5B). Figures SM5C and SM5D confirm that there is no overall effect of higher values for both CI and PE, except for CI from age 16, when both the overall and rate of change impact becomes bigger. This is visible in the hypothetical cases where there is absolutely no difference in CI in differing between skiers until the age of 16, when the skiers who score high and very high on CI start to take part in more competitions (Figure SM5E). In contrast, the PE hypothetical cases remain identical throughout the whole time.


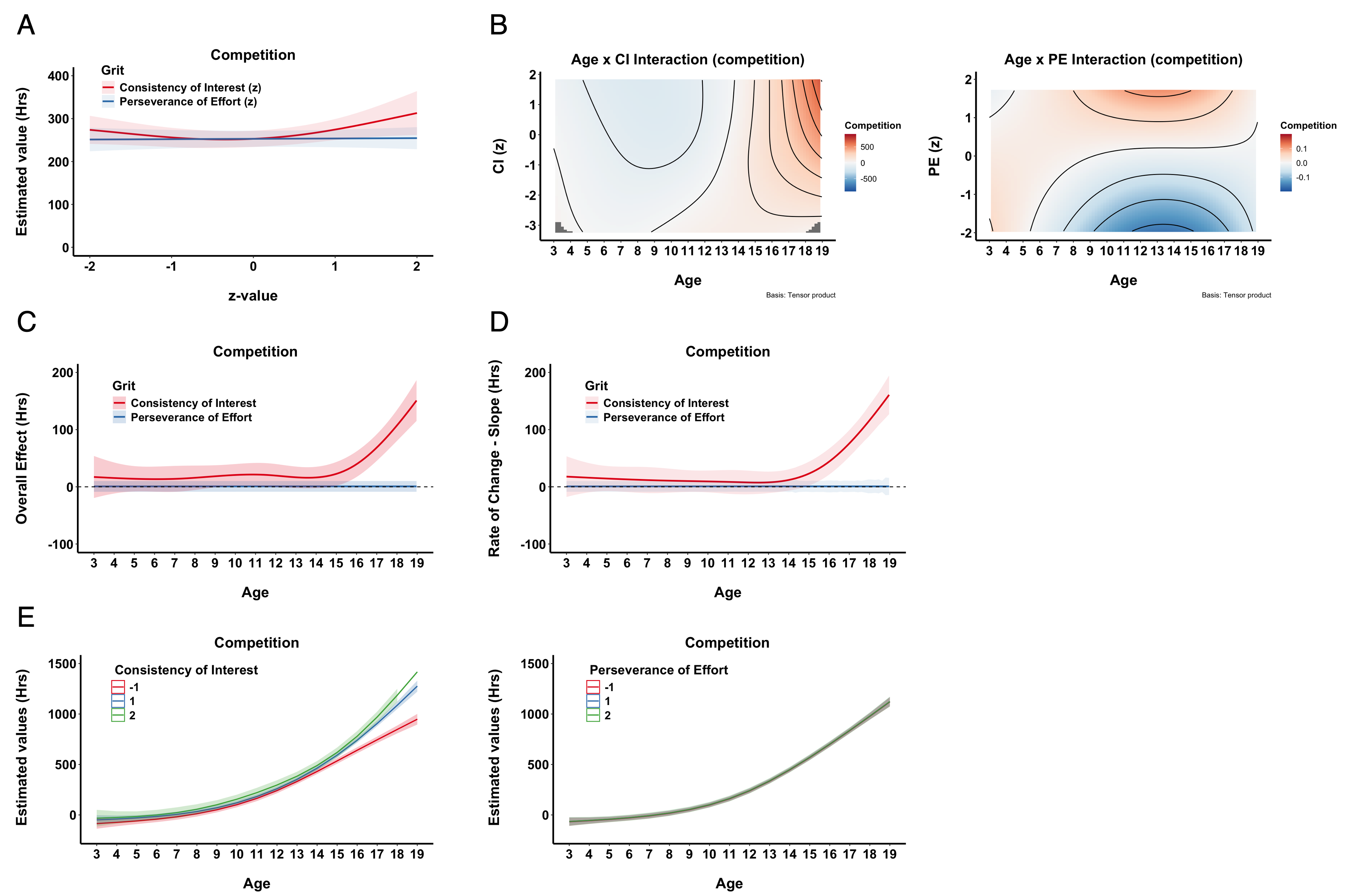


**Figure SM5. Competition (structured practice).** A) Predicted values across values of consistency of interest (CI) and perseverance of effort (PE). B) Interaction between age and CI (left) and PE (right) across age. C) Overall effects of CI and PE across age. D) The rate of change for CI and PE across age. E) Hypothetical practice curves over age for very high (+2z), high (+1z) and low (-1z) values for CI and PE. Dashed lines in C and D represent 0 or no effect. Shaded areas around the mean are +/- 1SE.

**Structured Practice – Coach Individual.** As in previous cases, the GAM model explaining the accumulated practice in individual sessions with a coach was significantly improved by adding CI and PE into the model (R^2^ = 0.24 vs. R^2^ = 0.20; F = 7, *p* < .001). Like other structured practice activities, CI and PE had little impact on taking part in individual training with a coach (Figure SM6A). If anything, the high CI individual tended to take part less in such activities. The influence over years was also more likely to decrease for both, but especially for CI (Figure SM6B). Figures SM6C and SM6D confirm the observation that from around age 10, both CI and PE decrease in influence, particularly the CI. The hypothetical cases (Figure SM6E) demonstrate this trend visually – the low CI skiers acquire more individual coach training than high CI skiers, with the difference being particularly pronounced in the later years. At the same time, differing values of PE have no impact on the acquisition curves for individual coach practice.


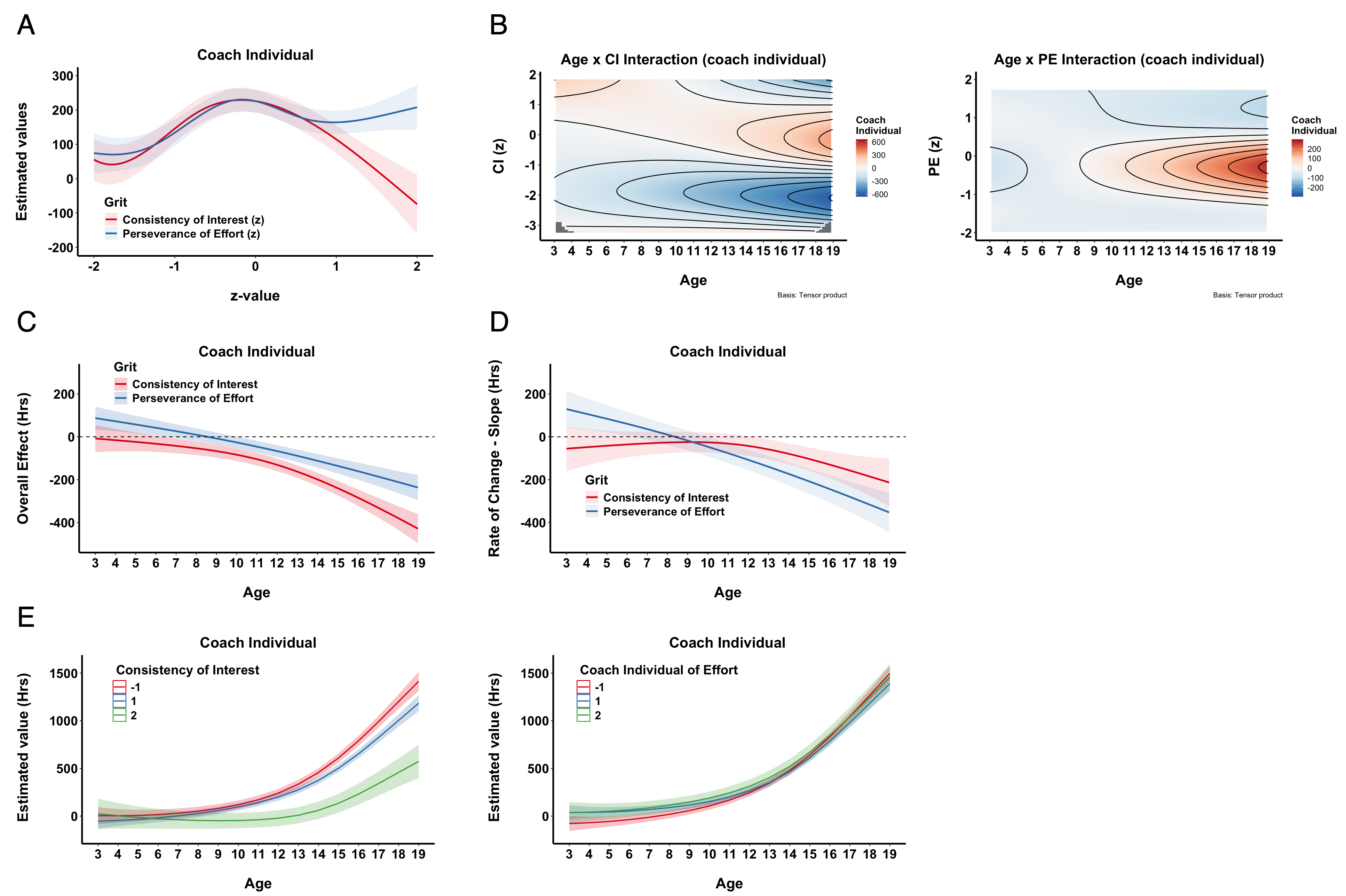


**Figure SM6. Individual Training with Coach (structured practice).** A) Predicted values across values of consistency of interest (CI) and perseverance of effort (PE). B) Interaction between age and CI (left) and PE (right) across age. C) Overall effects of CI and PE across age. D) The rate of change for CI and PE across age. E) Hypothetical practice curves over age for very high (+2z), high (+1z) and low (-1z) values for CI and PE. Dashed lines in C and D represent 0 or no effect. Shaded areas around the mean are +/- 1SE.

**Structured Practice – Coach Group.** Group practice with a coach was also better explained with than without CI and PE (R^2^ = 0.34 vs. R^2^ = 0.30; F = 6.9, *p* < .001). As with the individual coaching, CI even had a negative influence on the group training sessions with a coach, while PE this time had a slight positive influence (Figure SM7A). Both CI and PE saw their influence grow with age (Figure SM7B), but CI was becoming more and more negative, while PE had an increasingly positive impact (Figures SM7C and SM7D). This is clearly illustrated by the hypothetical cases where skiers with high CI acquire fewer group training hours throughout their young career than skiers with low CI (Figure SM7E). In contrast, the skiers with higher PE accumulate increasingly more group training hours as the time passes.


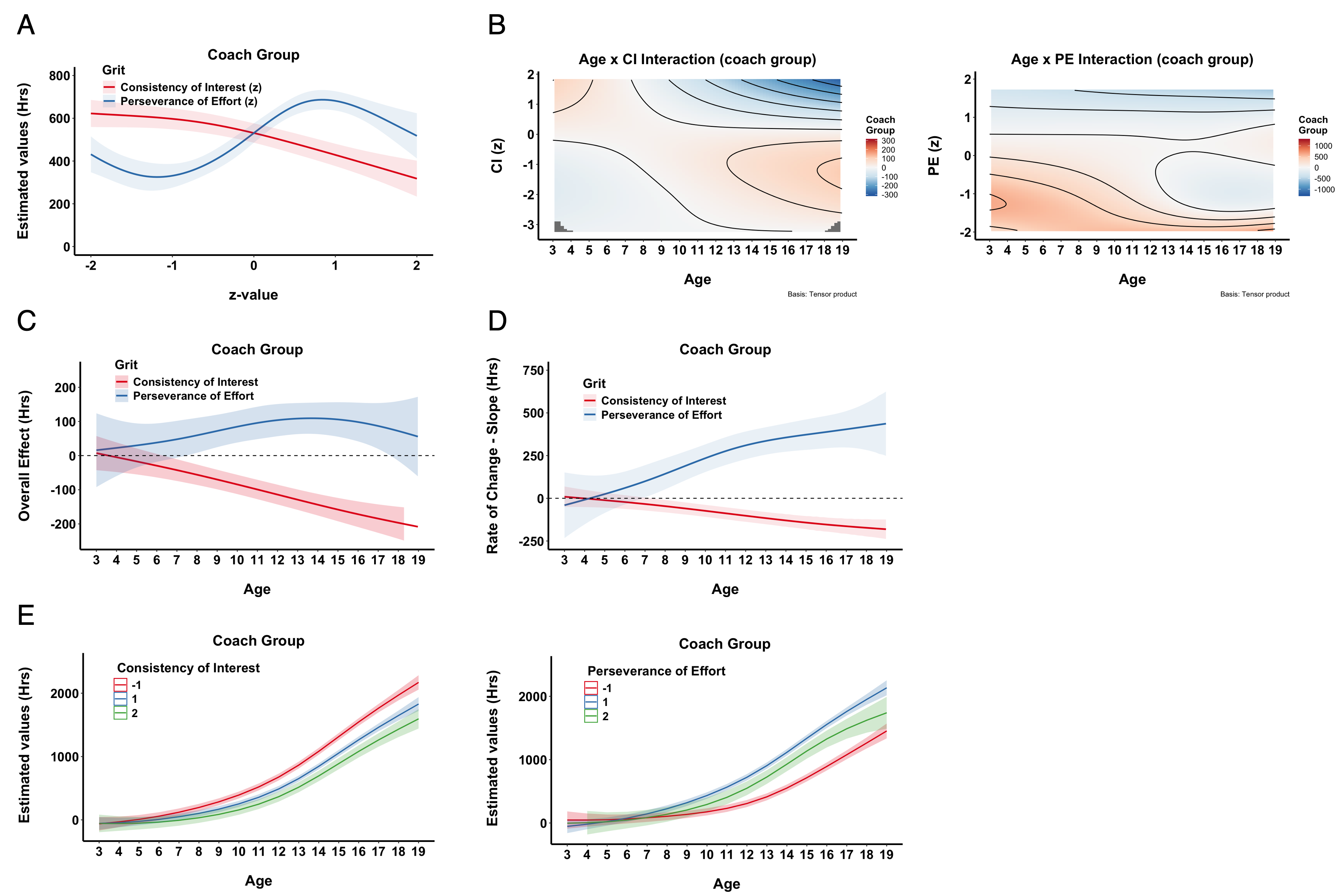


**Figure SM7. Group Training with Coach (structured practice).** A) Predicted values across values of consistency of interest (CI) and perseverance of effort (PE). B) Interaction between age and CI (left) and PE (right) across age. C) Overall effects of CI and PE across age. D) The rate of change for CI and PE across age. E) Hypothetical practice curves over age for very high (+2z), high (+1z) and low (-1z) values for CI and PE. Dashed lines in C and D represent 0 or no effect. Shaded areas around the mean are +/- 1SE.
